# Future tree survival in European forests depends on understorey tree diversity

**DOI:** 10.1038/s41598-022-25319-7

**Published:** 2022-12-01

**Authors:** Maik Billing, Kirsten Thonicke, Boris Sakschewski, Werner von Bloh, Ariane Walz

**Affiliations:** 1 Research Domain 1 “Earth System Analysis”, Member of the Leibniz Association, Potsdam Institute for Climate Impact Research (PIK), P.O. Box 60 12 03, 14412 Potsdam, Germany; 2 Institute of Environmental Science and Geography, University of Potsdam, Karl-Liebknecht-Str. 24-25, 14476 Potsdam-Golm, Germany

**Keywords:** Climate-change ecology, Ecological modelling, Forest ecology, Climate sciences

## Abstract

Climate change heavily threatens forest ecosystems worldwide and there is urgent need to understand what controls tree survival and forests stability. There is evidence that biodiversity can enhance ecosystem stability (Loreau and de Mazancourt in Ecol Lett 16:106–115, 2013; McCann in Nature 405:228–233, 2000), however it remains largely unclear whether this also holds for climate change and what aspects of biodiversity might be most important. Here we apply machine learning to outputs of a flexible-trait Dynamic Global Vegetation Model to unravel the effects of enhanced functional tree trait diversity and its sub-components on climate-change resistance of temperate forests (http://www.pik-potsdam.de/~billing/video/Forest_Resistance_LPJmLFIT.mp4). We find that functional tree trait diversity enhances forest resistance. We explain this with 1. stronger complementarity effects (~ 25% importance) especially improving the survival of trees in the understorey of up to + 16.8% (± 1.6%) and 2. environmental and competitive filtering of trees better adapted to future climate (40–87% importance). We conclude that forests containing functionally diverse trees better resist and adapt to future conditions. In this context, we especially highlight the role of functionally diverse understorey trees as they provide the fundament for better survival of young trees and filtering of resistant tree individuals in the future.

## Introduction

Climate warming threatens our forests and already increased tree mortality worldwide^1^. Forests are expected to undergo dramatic changes and losses, leaving the urgent question what helps forests to resist climate change in the twenty-first century^2^.

Biodiversity is considered as an important factor for stabilizing ecosystems^3,4^. Among the components of biodiversity, functional diversity—as the diversity of functional traits or groups—has shown to increase stability in plant ecosystems explained via various mechanisms: Functional diversity can lead to more efficient resource use, if groups or individuals strongly differ in their functional traits and complement each other (functional complementarity, Loreau and de Mazancourt^5^). This generally increases ecosystem productivity and overall individual survival helping the system to maintain functioning even under higher stress^6,7^. Additionally, functionally diverse ecosystems also exhibit broader response capacities to adapt to environmental changes. If one functional group fails, another can level out or replace its functioning, which is discussed in the light of the biological insurance hypothesis and port-folio theory^8,9^.

However, to date it remains unclear if and how potential positive effects of functional diversity observed today can help forests to resist climate change tomorrow^7^. Reasons for observed tree mortality or survival are likely to change in the future^10^, thus linkages between functional diversity and stability found under current climate conditions can be called into question. In addition, studies investigating functional complementarity and port-folio effects were mostly conducted in experimental grasslands, so that transferability to long-lived forests remains open^11–13^. Systematic long-term forest monitoring has a long tradition^14^. However, forest dynamics of the past may not be transferable to the current and future pace of change in light of global warming. Controlled manipulation experiments are urgently needed to understand forest response to changing environmental conditions, but remain challenging to implement and maintain, due to their constant need of financial resources, space requirements and large time horizons^15^.

Therefore, we require simulation models to assess what supports tree survival under climate change on decadal time scales and, more specifically, investigate the role of tree functional diversity to support forest resistance.

**Figure 1:**
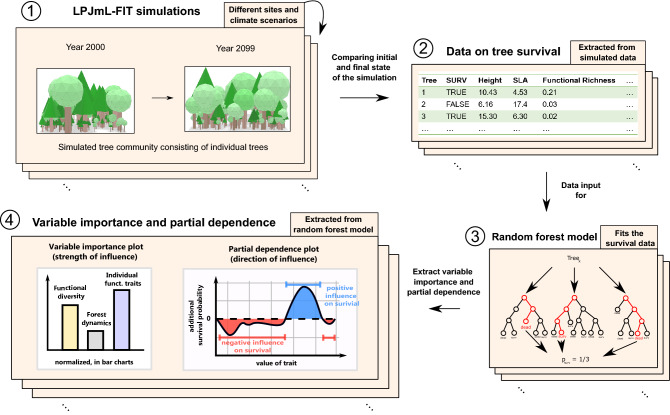
Summarizing scheme of the study design for different sites and climate scenarios. 1: LPJmL-FIT simulating individual trees applied to four European natural forests from year 2000 to 2099 under reference climate and future climate scenarios (here two exemplary snapshots from model output visualizations of Fig. 3—Supplementary Video 1). 2: Obtain data on tree survival from simulated tree community and on individual functional traits. 3: Initial forest conditions are used to predict tree survival probability through a random forest algorithm. 4: Variable importance and partial dependence plots are extracted from the random forest models for each variable and visualized in bar plots and line charts. A detailed description of the workflow can be found in Methods.

In this study we analysed the resistance of European natural forests under climate change using the next-generation Dynamic Global Vegetation Model LPJmL-FIT^16,17^. We refer to natural forests, as forests that are unmanaged for long periods and dominated by natural competition and regeneration. The model simulates individual trees with a flexible parameterisation scheme enabling to investigate the influence of tree functional diversity on forest resistance (see Supplementary Video 1). Hereby, we consider forest resistance as the ability of standing trees to survive under climatic changes. Each tree individual belongs to a plant functional type^18^ (thereafter PFT, involving: broad-leaved summergreen, broad-leaved evergreen, temperate needle-leaved and boreal needle-leaved trees). Leaf and stem functional traits can vary within a PFT. Tree survival depends on a mixture of environmental (e.g. climate) and competitive filtering related to natural forest dynamics. Our simulation experiments focussed on four natural to semi-natural Central European forest sites (an alpine needle-leaved forest, mixed mountainous forest, mixed temperate forest and a temperate broad-leaved forest; see “Methods” section, Supplementary Table S1) under 3 different climate change scenarios (RCP2.6, 4.5 and 8.5; Fig. 1, circle 1). To investigate if warming altered the influence of functional diversity on tree survival, we compare those future climate scenarios with simulations under a reference without climate warming (recycled climate between year 1951 and 1980; see simulation set-up under “Methods” section). To decipher which aspect determines forest resistance the most, we tracked the survival status of each simulated individual tree, its individual functional traits and respective environmental conditions including tree functional diversity as well as forest dynamic variables (Fig. 1, circle 2). Thereafter, we applied Machine Learning techniques (ML), i.e. random forest models to simulated individual trees (Fig. 1, circle 3), which allowed to determine importance and partial dependences of those aspects (Fig. 1, circle 4). Tree individual functional traits included specific leaf area (SLA, mm^2^/mg), wood density (WD, g/cm^3^) and tree height (m). Functional diversity was quantified by three established indices: Functional richness (FR), Functional divergence (FDv) and Functional evenness (FE). Forest dynamic variables included the number of locally competing trees > 5 m (ntrees) and local biomass as a proxy for the successional status (expressed as vegetation carbon, VegC in kgC/m^2^). A detailed description of the forcing data, site characteristics, the simulation protocol and of the data analysis can be found in the “Methods” section.

## Results and discussion

### Environmental and competitive filtering is most important for future tree survival

**Figure 2:**
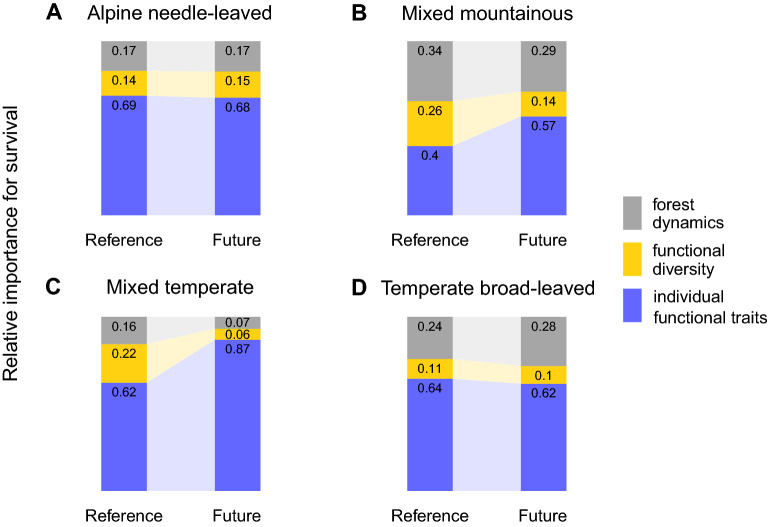
Relative importance of functional diversity, forest dynamics and individual traits for individual tree survival under reference climate and future climate conditions (RCP 4.5). Panels correspond to alpine needle-leaved (**A**), mixed mountainous (**B**), mixed temperate (**C**) and temperate broad-leaved (**D**) forests. Left bars in each panel illustrate reference climate (no warming) and right bars future climate (RCP4.5). Colours indicate forest dynamics (grey), functional diversity (yellow) and individual functional traits (blue). Forest dynamics include the number of locally competing trees (> 5 m in height) and local biomass as a proxy for the successional status.

We find that individual functional traits of each tree were most important for individual tree survival (40–87%) for all study sites, followed by forest dynamics (16–28%) and functional diversity (10–26%) (Fig. 2). Nevertheless, importance proportions substantially varied in each study site. While individual functional traits were least important in the mixed mountainous forest under reference climate (no climate warming), individual functional traits showed highest influence in the mixed temperate forest under future climate change (Fig. 2).

Tree survival depends on a mixture of environmental (e.g. climate) and natural competitive filtering, which excludes trees with trait combinations that underperform under local conditions^16^. Therefore, the high importance of individual functional traits across all study sites suggests a strong environmental and competitive filtering. Under future climate, the importance of individual functional traits generally increases or remains at high levels (Fig. 2). This shows that environmental and competitive filtering through functional traits are important processes to select best performing trees for the future, although being different for each forest type.

### Changing forest composition and trait shifts require large functional portfolio to secure forest resistance

**Figure 3:**
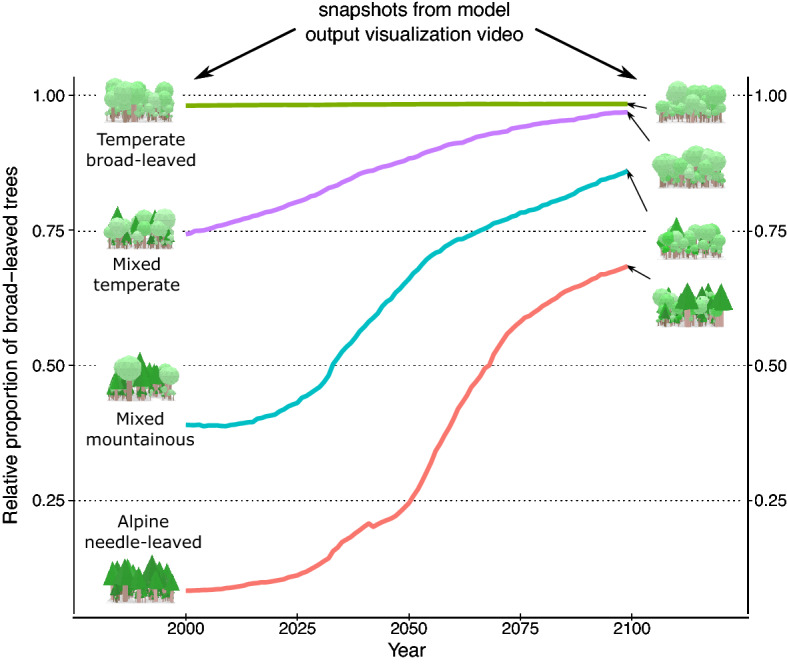
Forest compositions and changes in the proportion of broad-leaved trees (summergreen and evergreen plant functional type combined) under climate change (RCP 4.5) from 2000 to 2099 for each study site. The fraction of broad-leaved trees, as simulated by LPJmL-FIT for each site, increases gradually in almost every forest type reaching at least about 70% by the end of the century. Pictures depict snapshots from visualization of model output in the years 2000 and 2099, respectively. For a full animation of all sites from 2000 to 2099 please see Supplementary Video 1.

**Figure 4:**
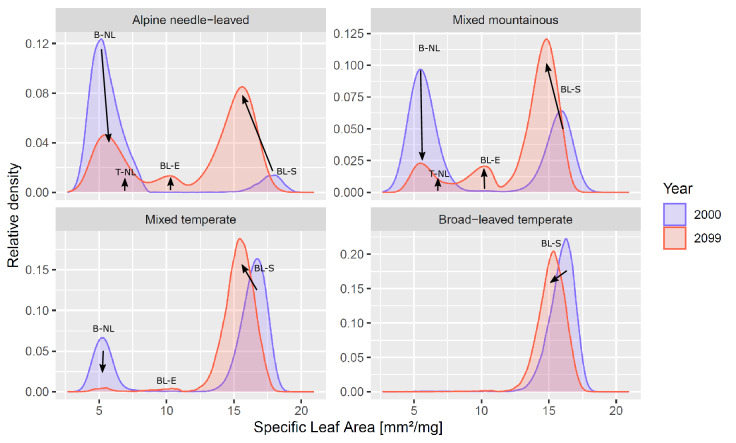
Trait distributions of specific leaf area in year 2000 and 2099, respectively, under future climate change (RCP 4.5). Arrows indicate trait shifts within plant functional types: *BL-S* Broad-leaved summergreen, *BL-E* Broad-leaved evergreen, *T-NL* Temperate needle-leaved, *B-NL* Boreal needle-leaved. For more detailed distributions see Supplementary Figs. S3 and S4.

Under future climate, we observe trait shifts within plant functional types and strong changes of the forest composition (Figs. 3, 4). Especially in the alpine and mixed forests, the proportion of broad-leaved trees increases to at least ~ 70% towards the end of the twenty-first century (Fig. 3). The changing climate alters environmental and competitive filtering simultaneously, whereby broad-leaved trees become more productive, survive better and increasingly outcompete needle-leaved trees. For instance, in the two mixed forests survival probabilities of broad-leaved trees (high SLA) increase by about ~ 10%, whereas the survival of needle-leaved (low SLA) trees is reduced by 10% to 30% in a warmer climate (see Supplementary Figs. S7, S8, Panel A). Locally better adapted and competitive broad-leaved trees can replace needle-leaved trees if they die and secure the forest’s overall biomass in the future. Nevertheless, our simulated forests still contain significant amounts of needle-leaved trees in the year 2099 in the two coldest study areas (Fig. 3, red and blue lines). Therefore, mixed tree communities with high functional diversity, where broad- and needle-leaved trees coexist, contain a broad range of functional niches out of which the best suitable plant strategies emerge and result in better resistance to climate change.

Simultaneously, we observe strong trait shifts in SLA within plant functional types across all study sites under climate change (Fig. 4). In general, the community of broad-leaved trees shift to lower SLA, while boreal needle-leaved trees are strongly reduced or slowly replaced by their temperate equivalent with higher SLA (Supplementary Fig. S3A, dark blue colours). In contrast, wood density distributions remain relatively broad and do not shift strongly under climate change (Supplementary Fig. S4). Throughout the century, the increasingly warmer climate filters new trait combinations leading to changes in the community composition within and across PFTs (see Supplementary Discussion B). Those trait shifts emerge from changes in the composition within PFTs and newly establishing PFTs, and could be less drastic if trait adaptation of tree individuals was considered (see “Limitations and outlook” section). The points raised above show, that trait ranges within and between PFTs should be wide to cover potential future trait shifts that secure future forest resistance.

All this suggests that functionally diverse forests are more resistant to future climate changes, due to their rich portfolio of traits. Broad trait distributions both within and between PFTs form the fundament for environmental and competitive filtering to select the most productive trees, securing the forest’s overall biomass under changing conditions. But can functional diversity further strengthen forest resistance beyond portfolio effects?

### Functional complementarity helps young trees to survive

We find that, in addition to port-folio effects, functional diversity increases forest resistance by supporting the survival of young trees to changing climate conditions via trait complementarity. Our results indicate, that trees benefit from functional diversity if they grow in tree communities with high FR, high FDv and low FE (Supplementary Figs. S6–S9, Panel D–F in each figure). Here, functional traits lay highly separated (FDv and FE) and span a broad range in the functional trait space (FR), enabling functional complementarity. Under these conditions the survival of trees increases up to + 16.8% (± 1.6%) depending on the study site and climate (Table 1). This effect is highest in the alpine and mountainous forests (14–17%), whereas it is less prominent or has an opposite effect in the two temperate forests (− 7% to 6%). That suggests, that complementarity effects are stronger in cold-limited and mixed forests where a marked cold winter season fosters a co-existence between broadleaved and needle-leaved trees. Both PFTs are specialized in fixing carbon during different times of the annual cycle: Due to their leaf phenology, needle-leaved trees can already be productive when broad-leaved trees are still in progress of unfolding or shedding their leaves. On the other hand, broad-leaved trees are more productive than needle-leaved trees during warmer months. If coexistence is given, these phenological differences enable complementarity and reduce competition among PFTs. That overall increases tree survival, because trees can invest more carbon in their stems and defensive structures if competition is lower. Therefore, we argue that phenological complementary can enhance tree survival and thus forest resistance. An in-depth discussion of those mechanisms is further found in Supplementary Discussion A.

**Table 1:** Additional survival probabilities for trees in each forest site under reference climate (central column) and future climate (RCP4.5, right column) in case FR and FDv are high, while FE is low.

Forest type	Mean additional tree survival probability from functional diversity	
	Reference climate	Future climate
Alpine needle-leaved	+ 16.5% (± 1.5%)	+ 16.8% (± 1.6%)
Mixed mountainous	+ 15.4% (± 1.8%)	+ 13.7% (± 1.0%)
Mixed temperate	− 7.1% (± 1.1%)	+ 1.6% (± 1.0%)
Broad-leaved temperate	+ 6.7% (± 2.2%)	+ 4.8% (± 1.1%)
Mean	+ 7.9% (± 0.8%)	+ 9.2% (± 0.6%)

Survival odds from partial dependence plots were extracted for high FR, high FDv and low FE (upper and lower 10%-percentiles) separately and summed up based on Supplementary Table S3. Values in brackets correspond to error propagated standard deviations from Supplementary Table S3.

**Figure 5:**
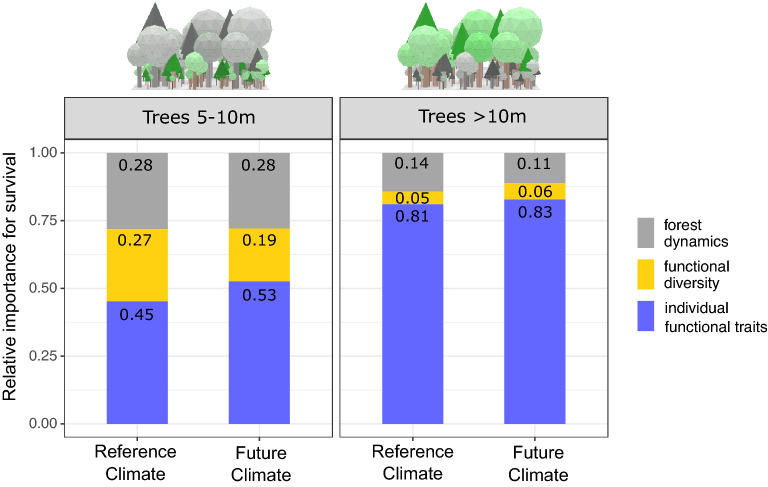
Variables importance for small and large trees. Normalized variable importance for forest dynamics (grey), functional diversity (yellow) and individual functional traits (blue) averaged across all forest types under reference climate (left bars) and future climate (RCP 4.5, right bars) for small trees (5–10 m) in left panel) and large trees (> 10 m) in right panel, respectively. Functional diversity and forest dynamics are more important for small trees compared to large trees, whereas individual functional traits matter most for large trees. This pattern was found to be consistent across all sites (see Supplementary Table S6).

Surprisingly, our results show that those complementary effects are much more important for small trees (< 10 m) to survive: The importance of functional diversity accounts for ~ 20% among trees smaller than 10 m, while it is negligible for trees above 10 m (Fig. 5). Competitive filtering is generally stronger if trees are small and grow more compactly. Therefore, complementary effects—which can lower competition—are particularly stronger and therefore more important. Once trees grow higher and reach the canopy, tree survival increasingly depend on individual performance under the given climate and thus individual functional traits gain importance. Similar effects in colder forests were also found in other studies^19^, where functional diversity increased the productivity of smaller trees in cold and temperate forests. In summary, we find that the functional diversity of the entire tree community can support especially understorey tree survival. Above all, we observe that effects of functional diversity are positive under future climate—however different for each study site (from 1.6 to 16.8% Table 1). This underlines the importance of functional complementarity to support understorey tree survival in the future and indicates, that positive effects of functional tree trait diversity also persist under climate change.

### Functionally diverse understoreys unlock the synergy of filtering and complementary effects

Our findings underline the role of functionally diverse trees in the understoreys for forest resistance. On the one hand, functional diversity supports the survival of understorey trees via functional trait complementarity. On the other hand, they form the fundament for competitive and environmental filtering. Only diverse tree communities have trait pools large enough to ensure that their tree portfolio holds trait combinations best suited for changing climate conditions. Therefore, we argue that functional diversity does not only support tree survival through complementarity, but is a prerequisite for filtering resistant trees in the first place.

To profit constantly from functional diversity of the understory and ensure constant adaptation, a diverse age structure is a prerequisite. Depending on the forest type, trees are distributed in a broad range of different height and age classes in our study (Supplementary Fig. S5, Supplementary Video 1). This multi-aged structure is preserved under climate change (Supplementary Fig. S5) and allows gradual changes through constant environmental and competitive filtering in the future.

In this study, we simulate forests without any human interference or management. Our results are therefore to be interpreted in the context of environmental and competitive filtering as observed in natural forests. Most managed forests lack this natural filtering effect as they are less dense and diverse in their age-structure. Functionally diverse trees in the understorey could provide the fundament for climate adapted multi-aged forests, as they constantly form new better adapted tree generations with natural competition and succession allowed. Therefore, we fully underline the importance of functionally diverse understorey trees *and* natural competition as the fundament for future forest resistance.

### Management implications

The results of this study highlight the importance of functionally diverse understorey trees. However, browsing by game might damage new tree saplings and limit tree diversity in the understorey. In addition, invasive species like *Prunus serotina* or herbaceous competition might hinder forest succession and the establishment of woody native species in European forests^20,21^. Therefore, regulating game, limiting the spread of invasive species and controlling herbaceous vegetation should be considered in future management practices where tree diversity in the understorey seeks to be increased or maintained. Moreover, insufficient dispersal of functionally different tree species might limit the establishment of functionally diverse trees in the understorey. Future forest management may consider to artificially plant functionally different tree species if dispersal from surrounding forests cannot be guaranteed. On the other hand, forests that already contain functionally diverse trees in the understorey should be preserved.

In this study, a clear trend from needle-leaved to broad-leaved trees is captured at all sites, whereas within broad-leaved PFTs a shift to lower specific leaf area and higher leaf longevities indicates that future forests might especially benefit from longer vegetation periods (earlier leaf onset, later senescence). Therefore, forests containing broad-leaved tree individuals with high phenological plasticity could be more resistant. The broad simulated wood density ranges, which persist under climate change, imply beneficial effects for forest communities entailing a range of different growing strategies, i.e. early to late successional species. Therefore, we argue that forest fragmentation should be reduced or reversed to foster some natural dispersal of early and late successional species.

This study intended to explore the potentials of functionally diverse forests as a possibility to stabilize forests under climate change over a large climatic gradient. The model used in this study operates on the more general level of functional traits and their diversity rather than on species level (see “Methods” section and Supplementary Methods A). Consequently, management implications regarding suitable specific tree species are beyond the scope of this study. However, we think that our results will stimulate the discussion on the importance of functional tree traits and their diversity for species selection.

### Limitations and outlook

This study focussed on identifying the importance of functional diversity for future tree survival to advance our understanding on the role of biodiversity for future forest resistance using the flexible-trait Dynamic Global Vegetation Model LPJmL-FIT. The general approach of LPJmL-FIT is to simulate biogeographic dynamics purely based on environmental and competitive filtering (see “Methods” section, Supplementary Methods A). Due to missing processes in the model and the ambiguity of former human influence, drawing site-specific implications on future forest dynamics must be taken with caution (see Supplementary Discussion D).

Moreover, processes not yet captured in the LPJmL-FIT model, might play a role and could lower forest resistance in the future, which is why we recommend relying on a trait space as broad as possible.

Including more climate scenarios would widen the envelope of possible future pathways by considering climate model uncertainties. Insect outbreaks and pathogens might put pressure to the already drought- and temperature-stressed trees and heavily accelerate mortality especially of needle-leaved trees, although functionally diverse forests are less vulnerable to bark beetle outbreaks^22,23^. Multi-layered forests showed higher growth resilience to structural disturbances such as wind-throw^24^, likely enhancing the importance of individual tree height and reduce the survival probability of large trees^25^. Belowground competition and trait plasticity could favour complementarity effects further. Variable rooting strategies could further reduce competition for soil water and thereby increase individual drought resistance of trees^26^. Trait plasticity can contribute to tree survival by widening niche, further increasing complementarity effects. However, trait plasticity remains one of the most challenging objectives in vegetation modelling as observational data and modelling approaches are scarce^27^, leaving it open how far trait relationships would hold under climate change. Considering more functional traits in our analysis might increase the overall predictive power of the random forest models. Even though the explained variance increased with the number of analysed traits explaining ecosystem properties in long-term grassland experiments, such improvement is limited as abiotic factors and their interactions with plant traits might be more important for prediction^28^. We conclude that simulating future forest dynamics dominated by environmental and natural competitive filtering requires to integrate both, abiotic and biotic drivers on forest dynamics. Machine learning techniques are increasingly used in forest ecological research—but mainly applied in the processing of field and forest inventory data^29,30^. Machine learning can help to understand the complexity of interactions and provide deeper insights into the underlying ecological process in a modelling study as we have shown here using LPJmL-FIT simulation results. Random forest analyses are suitable for a variety of data and applications because they are relatively robust to different data structures. Importance analysis can help to identify the role of underlying processes in complex models and to visualize their changes in a simple way. In doing so, model development is advanced by making use of large data sets, opening the door to further theory building and deeper understanding of plant trait ecology.

## Conclusion

The present study investigated if and how functional diversity of trees can help European forests to resist climate change. Therefore, we analysed the importance of individual functional traits, functional diversity and forest dynamics for individual tree survival under present and future climate in a flexible-trait Dynamic Global Vegetation Model. In general, we find that forests benefit today and under climate change from tree functional diversity. Individual traits were most important for overall tree survival (40–87%) indicating that environmental and natural competitive filtering is crucial to future forest resistance. Future trait shifts and changes in forest composition require large functional portfolios to filter best adapted trees. Trait shifts indicate that tree individuals with high phenological plasticity could be more resistant in the future. The importance of functional diversity appeared relatively high for trees in the understorey (~ 25%) and functional diversity supported tree survival of trees in the understorey by up to + 17% (± 1.6%) due to complementary effects, which persist under climate change. Therefore, we highlight the synergy of functional trait complementarity *and* natural competition for future forest resistance. We conclude that forests require functionally diverse trees in their understoreys as a basis to enable filtering and the support of understorey trees via functional complementarity.

## Methods

To investigate what controls the survival of individual trees under future climate, we applied the flexible-trait DGVM LPJmL-FIT. The main idea in the analysis was to compare changes in simulated forests at a certain point in time against its reference state in the year 2000. This allowed to assess which trees survived and which died, while simultaneously knowing the initial individual conditions of every tree and its surrounding community. Based on that information, we subsequently applied random forest models to simulated results to capture which features and processes mostly influence the survival of individual trees (Fig. 1). In the following, we describe the model and our simulation set-up, how we extracted the relevant data, applied random forest models to them and finally obtained importance measures and partial dependence plots.

### The LPJmL-FIT model

We applied the flexible-trait DGVM LPJmL-FIT^16,17^ to four different sites in central Europe. LPJmL-FIT simulates explicitly individual trees and grasses following the concept of Plant Functional Types (PFT)^18^. Trees belong to one out of four woody PFTs (broad-leaved summergreen, broad-leaved evergreen, temperate needle-leaved, and, boreal needle-leaved trees). Grasses are simulated on the basis of two herbaceous PFTs (temperate C_3_-grasses and tropical C_4_-grasses) as in earlier publications^16^. The model simulates natural forest dynamics, including hydrology and carbon cycle. In each simulation step, trees and grasses compete for light and water on patches of 10 m × 10 m size. The general approach of LPJmL-FIT is to simulate biogeographic dynamics based on environmental and competitive filtering without using observational data of real forest communities to initialize the model. As the general approach, every tree growing strategy can establish in every grid cell at any time, while only environmental and competitive conditions determine which tree individuals survive over time and which do not. Therefore, the model is only driven by climate and soil data. Simulated tree communities are a model output and can be compared to real forest communities (see Supplementary Table S1, Supplementary Discussion D for validation).

**Figure 6:**
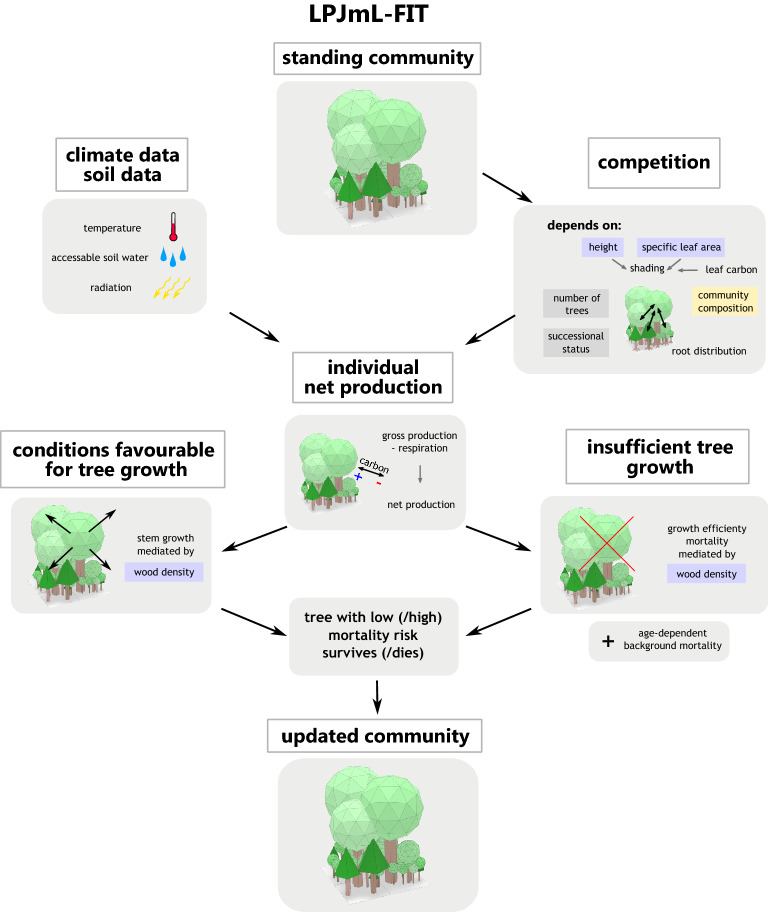
Scheme of central mechanisms of the LPJmL-FIT modelling approach. Local competition, climate input, and individual functional traits determine the net production of every tree individual. Tree survival depends on tree age and the individual carbon balance mediated by wood density. A more detailed description is found in Supplementary Methods A–C.

In LPJmL-FIT, each tree is characterized by PFT-specific phenology and a unique combination of leaf and stem functional traits, which are connected via empirical derived relations based on the leaf and stem economic spectrum^17^. Traits and phenology parameters are assigned randomly during the establishment of a new tree and remain fixed during the whole lifetime of the individual (except tree height). Traits were drawn out of PFT-specific ranges according to global plant trait databases^31^. Due to environmental filtering from competition among trees and the local climate, only trees with locally best-suited trait combinations survive (Fig. 6). Through this flexible-individual trait approach, tree communities can be visualized as trait clouds in multi-dimensional traits spaces illustrating the occupation of ecological niches. Therefore, the model provides information about the trait composition of the whole tree community, from which functional diversity indices were computed following the methods described in earlier publications^16^. The model has been extensively validated on biomass, tree height, gross primary production and trait distributions^16^ as well as tree mortality (Supplementary Methods C). Additional model description is found in Supplementary Methods A and earlier publications^16,17^.

### Model input data and site characteristics

The model is driven by climate (mean daily temperature, precipitation and radiation), atmospheric CO_2_ concentration and soil data. We used daily climate data from Hadgem2-ES^32^ bias-corrected with WATCH data^33^ on a 0.5° × 0.5° longitude-latitudinal grid ranging from year 1951 to 2099 for three different RCPs (RCP2.6, RCP4.5, RCP8.5) and held the atmospheric CO_2_ concentration constant at 296 ppm over the whole simulation period. The fixed CO_2_ concentrations account for limitation effects not considered in LPJmL-FIT. For all grid cells the soil depth was set constantly at 2 m and soil texture was used from the Harmonized World Soil Database (HWSD) version 1.2^34^. We applied the model to four different sites containing near-natural forest stands, which are located in the protected areas Kalkalpen (Austria), Laegern (Switzerland), Hainich (Germany) and Bialowieza (Poland). The sites cover a wide range of central European climates and were subsequently named according to their simulated forest compositions and characteristics: alpine needle-leaved, mixed mountainous, mixed temperate and broad-leaved temperate forest. For detailed site descriptions and model validation at site level, see Supplementary Table S1 and Supplementary Discussion D.

### Simulation set up

We started the model simulation at each forest site from the bare-ground and recycled the first 30 years of the input climate data (1951–1980) for a total period of 500 years bringing the simulated vegetation into equilibrium (spin-up). Starting from the spin-up model states, we performed four different simulation experiments under transient climate at each forest site: (1) the reference climate experiment without climate change, where the spin-up climate covering 1951 to 1980 was shuffled and repeated until 2099, and (2) three climate change experiments, one for each RCP (2.6, 4.5 and 8.5) covering 1951 to 2099 (see Supplementary Fig. S1 for projected climate changes in each study site). At each site we simulated 10.000 independent patches of 10 m × 10 m, which corresponds to a total forest area of 100 ha. Rooting depth of each tree was limited to 3 m for every simulation cell and the vertical distribution fine roots across soil layers was hold constant for each PFT. A visualization of model simulations at each study site (each 25 forest patches) between years 2000 and 2099 under RCP4.5 is provided in Supplementary Video 1.

### Data analysis

All data analyses were conducted using the free programming language R v3.6^35^. For each model run we extracted all simulated trees larger than 5 m in the year 2000 and in the year 2099, respectively. Trees < 5 m were regarded as saplings, and thus excluded from the analysis. By comparing these two time slices, we assessed which trees, that are present in year 2000, survived and were still present in 2099, and which had died (Fig. 1, circle 1).

Further, we combined this data on individual tree mortality with information on individual functional traits, functional diversity and forest dynamic variables extracted from the simulation state in year 2000 (Fig. 1, circle 2). Individual functional traits include specific leaf area (SLA, mm^2^/mg), wood density (WD, g/cm^3^) and tree height (m), whereas forest dynamic variables involve number of standing trees > 5 m (ntrees) and local biomass (expressed as vegetation carbon, VegC, kgC/m^2^) as a proxy for the current successional status. Functional diversity is quantified by three established indices, which capture different aspects of functional diversity. Functional richness (FR) specifies the extent of the occupied trait space. Functional divergence (FDv) indicates how far abundance distribution in niche space deviates from the centre and specifies the degree of niche differentiation. Functional evenness (FE) illustrates niche separation and efficient resource use which is important for plant co-existence^16^. We adopted the computation of FDv, FR and FE from earlier publications, where a detailed description of the indices can be found^36,37^. Functional diversity indices were based on SLA, leaf longevity and wood density and were calculated for each patch independently. We based the calculation of the indices on these three traits, because they strongly determine tree performance and general model behaviour, and can be considered as central traits in the leaf and stem economic spectrum (see also Supplementary Methods B). In this study, the distribution of the FR showed a strong positive skewness, which was reduced by taking its natural logarithm (ln(FR)).

Thereafter, we build conditional random forest models (RF hereafter) using the “cforest” function in R-package “party”^38^ to predict the individual survival probability of trees standing in the year 2000 depending on individual functional traits, forest dynamics and functional diversity in year 2000 (Fig. 1, circle 3). Because tree mortality rates were systematically different across forest types, we subsampled equal numbers of dead and living trees to increase comparability across forest types and reduce potential sample biases in the RFs, resulting in a total number of 5000 trees for each run and forest type. We split the raw data into training data (80%) and test data (20%) sets and performed the tuning of the hyperparameters ntree (number of trees in the RF) and mtry (number of split variables). Therefore, we built RFMs using the training data set and evaluated the AUC (receiver operator characteristic area under the curve) of the test data for model quality assessment. We variated ntree from 50 to 800 in steps of 50, leaving mtry constant at 2. This parameter variation showed that ntree = 500 was optimal, while higher ntree led to no further model improvement (Supplementary Fig. S10). Subsequently, the hyperparameter mtry was varied from 2 to 8 with constant ntree = 500. Here, mtry = 3 led to the best results in almost all cases (Supplementary Fig. S11). Consequently, we chose ntree = 500 and mtry = 3 for our main analysis across all study sites. We rebuild all RF for all runs und input climates using the training data. Characteristics (mean, standard deviation, moments, QQ-plots) of the training data are provided in the Supplementary Fig. S12 and Supplementary Table S4. Final RF model quality was evaluated on the basis of AUC and accuracy (ACC) in relation to the test dataset and ranges in an acceptable manner between 0.60 and 0.76 for accuracy and 0.63 and 0.87 for AUC depending on the forest type and input climate (Supplementary Table S5).

In a next step, we calculated the permutation importance of the mean decrease in accuracy using the “varimp” function of the R-package “party”^38^ (Fig. 1, circle 3). In order to permit comparability among the RFs, we normalized the resulting variable importance values to one, resulting in normalized variable importance for each RF model. The importance rankings were robust under the repeated execution of the algorithms with varying random seed yet showing small stochastic fluctuations in absolute values. To reduce this variability, we repeated the calculations for 10 times deriving their mean value from them. Normalized variable importances were visualized (Fig. 1, circle 4) using the “ggplot2” package^39^. Those variables’ importance plots show how *strongly* each variable contributes to the predictability of tree survival. To analyse whether the variables’ importance changes depend on the height of a tree, we further split the tree community into small tress (< 10 m, denoted as trees in the understorey) and large trees (> 10 m). The threshold for separating the tree community into small and large trees was set to 10 m, because dependence plots showed shifting behavior at about 10 m tree height (either switch from positive to negative or/go into a plateau afterwards in Supplementary Figs. S6–S9), which might indicate different processes to explain the survival of trees above/below this value. For those two groups we recalculated the RF models and evaluated the variables importances separately.

For further analysis, we computed partial dependence plots (PDP) of each variable^40^ for the whole tree community. PDP illustrate the effect (positive or negative) of a single variable on the predicted survival probability assuming all other variables as hold constant. Therefore, we sampled 1500 trees out of the training dataset and fixed one variable to its particular value in the PDP. Thereafter, we predicted the survival probability according to the RF and averaged the survival probability over all trees. Thereby, a point in PDP can be interpreted as the single contribution of a variable to the overall survival probability assumed all other variables as distributed in the total tree population. Values above zero indicate that a certain variable value contributes positively to survival and vice versa (Fig. 1, circle 4). These PDP include *how* tree survival is controlled by certain traits, as they show whether a variable contributes positively or negatively to tree survival. All PDP can be found in the Supplementary Figs. S6–S9. Further, we estimated the combined effect of FD on tree survival. Therefore, we extracted the upper and lower 10% percentiles of the initial distributions (in the year 2000) of FD indices for each study site. Those percentiles corresponded to the upper and lower ends of the distributions. Subsequently, we averaged the partial dependence of each FD index over their upper and lower tails, respectively. Therefore, we obtained a measure for the additional odds to survival depending on the FD index (Supplementary Table S3). To estimate the overall effect of high niche separation (e.g. highly separated clouds in functional trait space), we summed up the cases for high FR, high FDv and low FE (Table 1).

In a next step, we extracted the temporal development of the above and belowground forest biomass (aggregated to vegetation carbon, VegC, kgC/m^2^), fractions of PFTs and trait distributions from the model simulations. Finally, we extracted the combination of all three components: we analysed variable importance, partial dependence and the temporal evolution of forest characteristics, which led us to a better understanding of the controlling ecological processes behind tree resistance in the model.

## Supplementary Information

Supplementary Information.

Supplementary Video 1.

## Data Availability

All data needed to evaluate the conclusions in the paper are present in the paper and the Supplementary Materials. The data can be provided by Potsdam Institute for Climate Impact Research (PIK e. V.) upon reasonable request and pending scientific review.

## Code availability

Programming code can be provided by Potsdam Institute for Climate Impact Research (PIK e. V.) upon reasonable request and pending scientific review.
