# Supplementary material for: Future tree survival in European forests depends on understorey tree diversity
Source: Sci Rep. 2022 Dec 1;12:20750. doi: 10.1038/s41598-022-25319-7 (PMC9715543; doi:10.1038/s41598-022-25319-7)
Supplement: Supplementary file 1 — Supplementary Information. [file 41598_2022_25319_MOESM1_ESM.docx]

# Supplementary Information

## Supplementary Figures and Tables


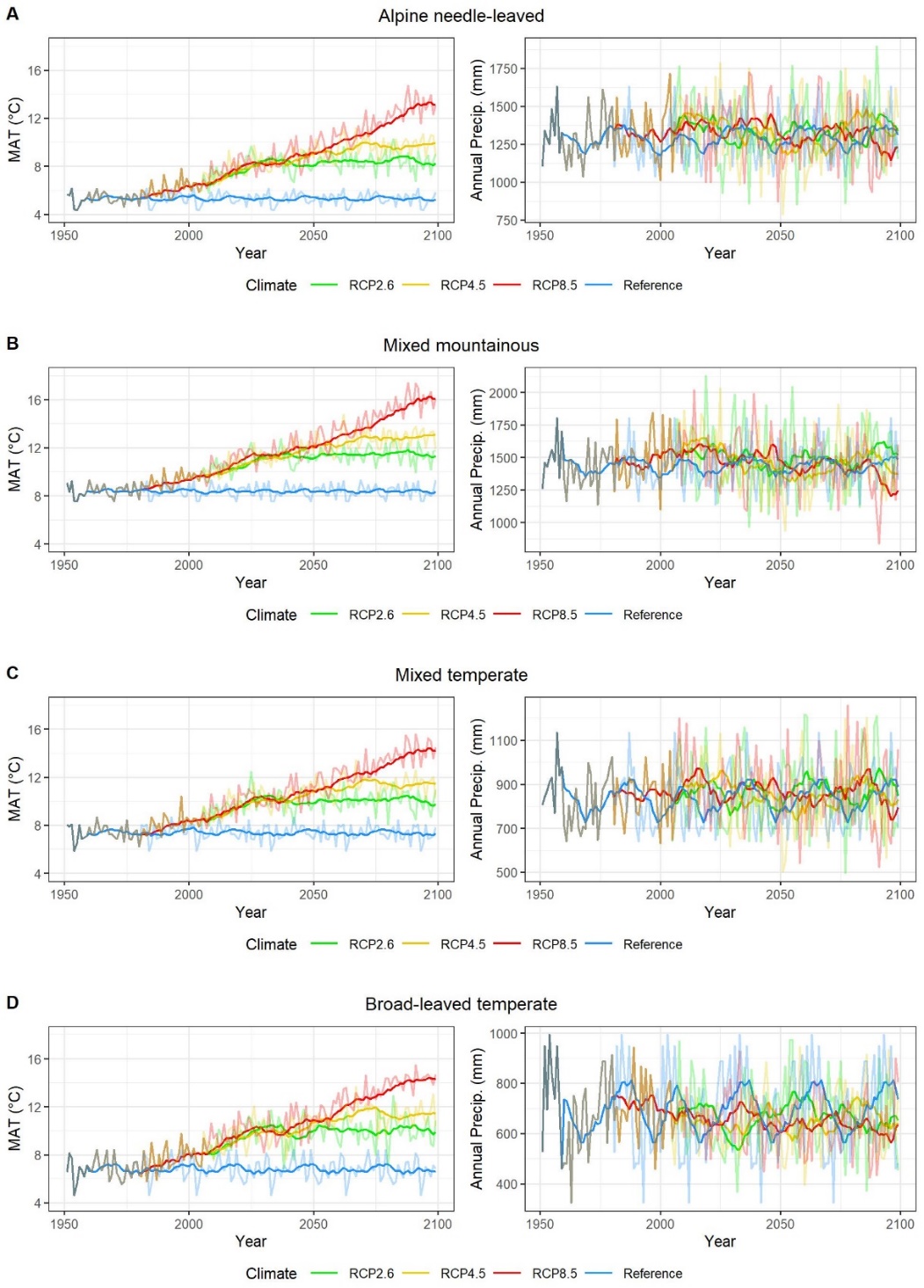


#### Supplementary Figure S1:

**Climate data across all forest types.** Mean annual temperature (MAT) and annual precipitation (Annual Precip.) for each RCP and the reference climate across all forest types. The reference climate (blue lines) is the 30a recycled climate data between 1951 – 1980. Opaque lines show the centred moving average over 10 years.

####
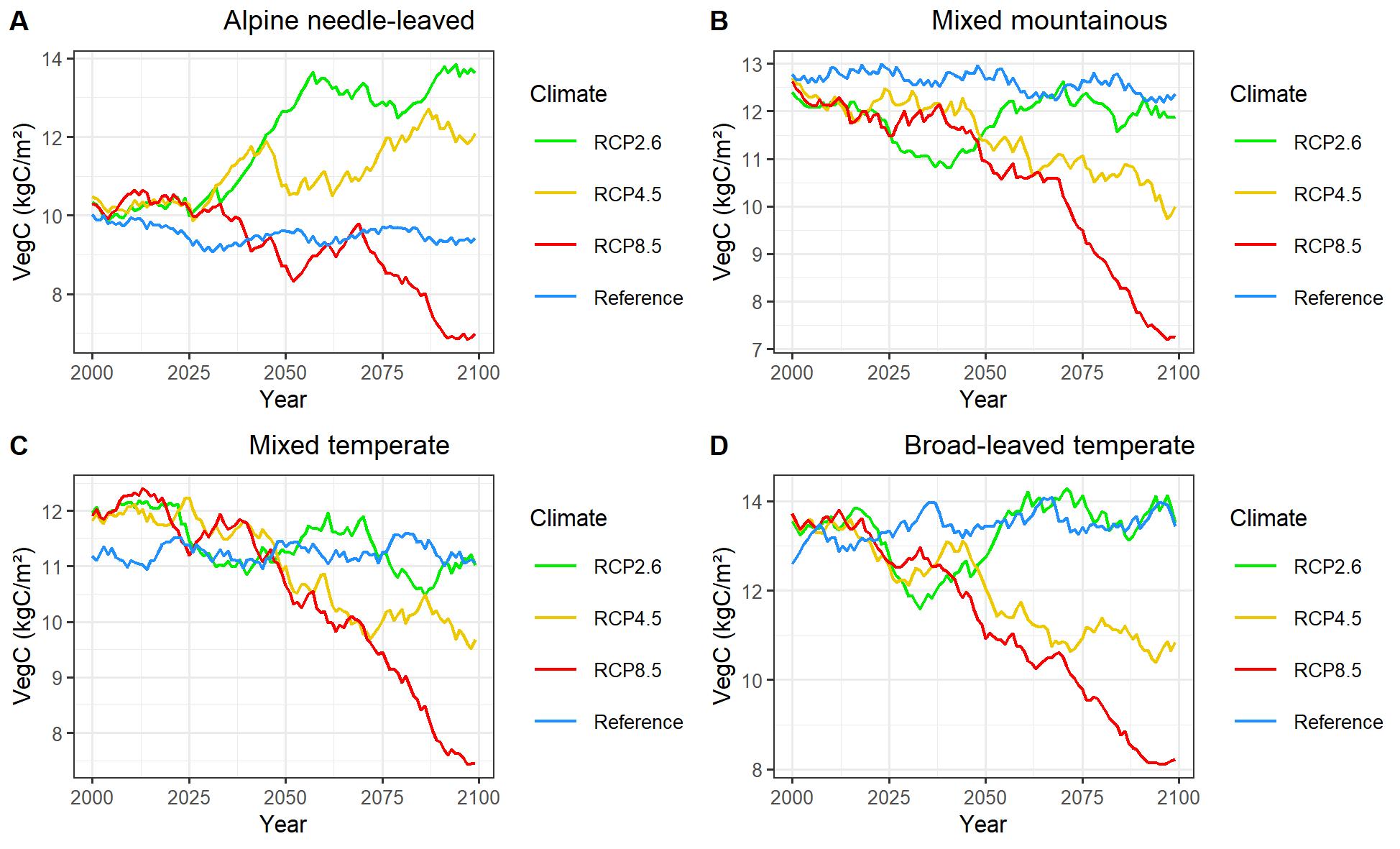
Supplementary Figure S2:

**Vegetation carbon (VegC).** Simulated above and belowground vegetation carbon across all forest types and input climates.


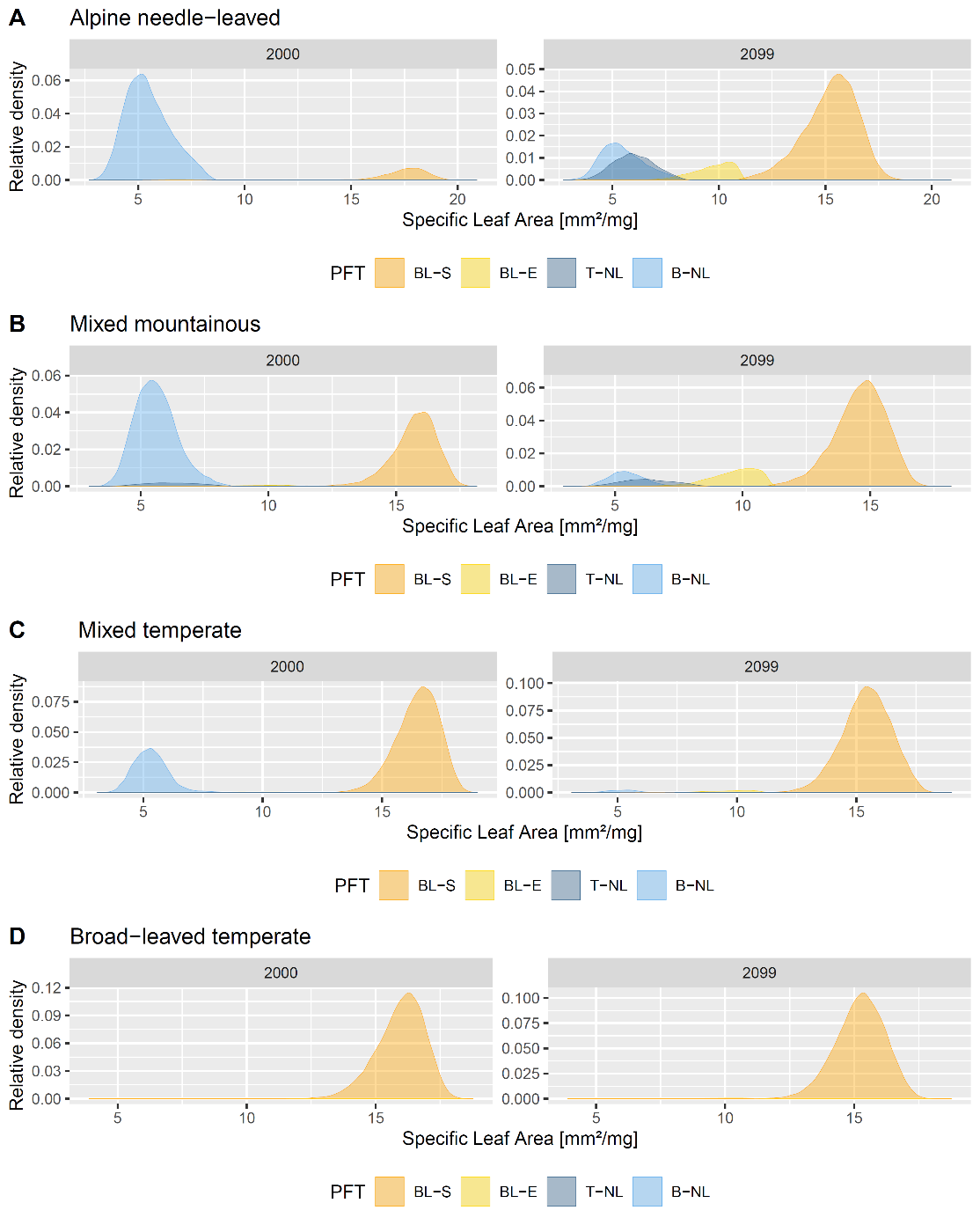


#### Supplementary Figure S3:

**Trait distributions of specific leaf area for each study sites.** Normalized distributions of simulated specific leaf area (SLA) in year 2000 and 2099 (under RCP 4.5) for each plant functional type: BL-S: Broad-leaved summergreen; BL-E: Broad-leaved evergreen; T-NL: Temperate needle-leaved; B-NL: Boreal needle-leaved.


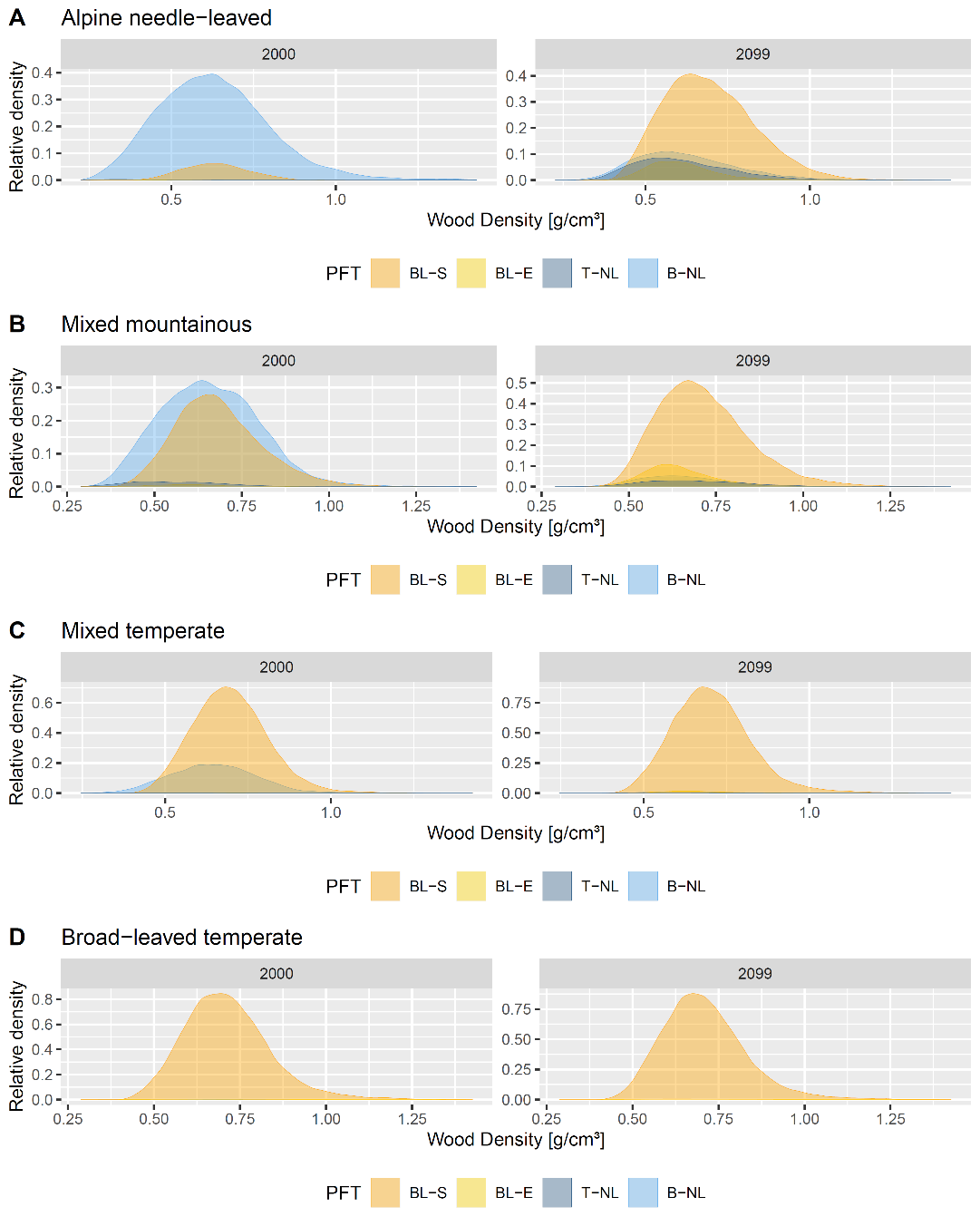


#### Supplementary Figure S4:

**Trait distributions of wood density (WD) for each study site.** Normalized distributions of simulated wood density (WD) in year 2000 (left side) and 2099 (under RCP 4.5; right side) for each plant functional type: BL-S: Broad-leaved summergreen; BL-E: Broad-leaved evergreen; T-NL: Temperate needle-leaved; B-NL: Boreal needle-leaved.


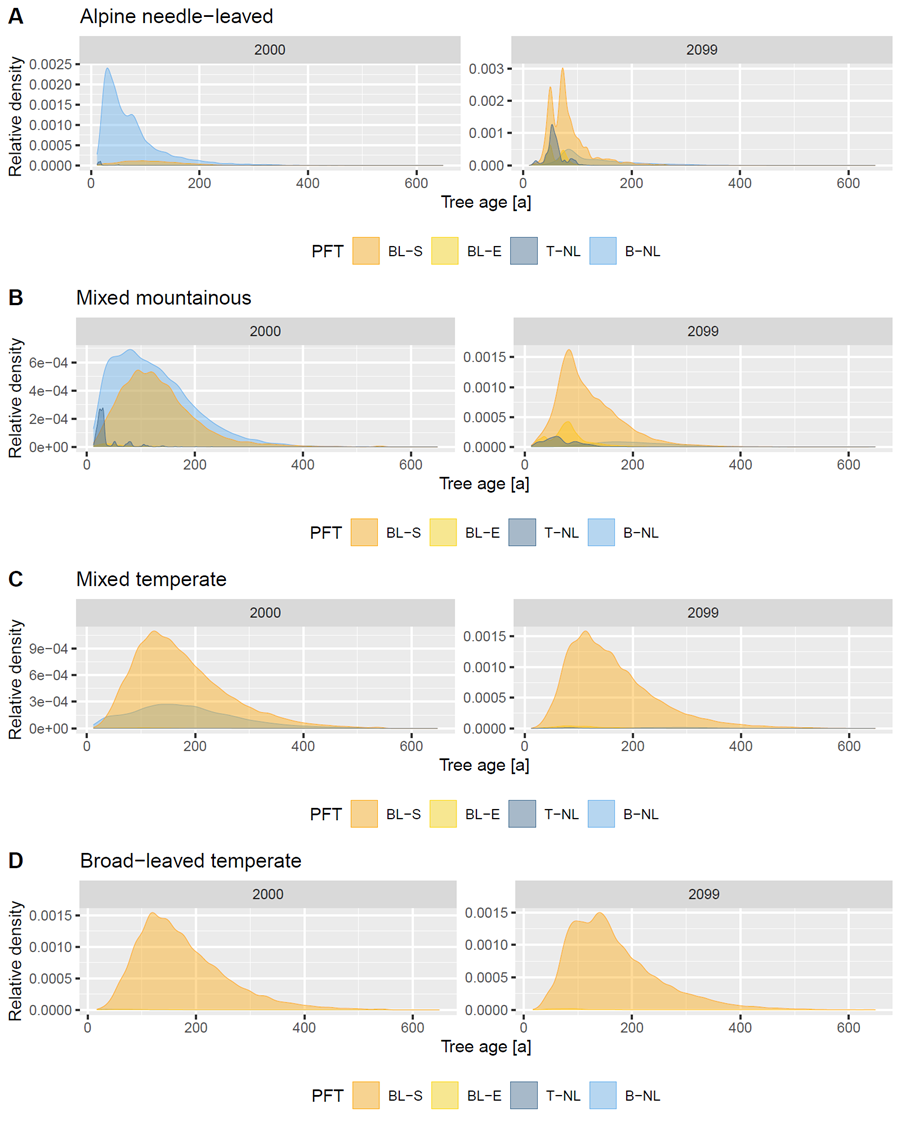


#### Supplementary Figure S5:

**Distributions of tree ages for each study site.** Normalized distributions of simulated tree age in year 2000 (left side) and 2099 (under RCP 4.5; right side) for each plant functional type: BL-S: Broad-leaved summergreen; BL-E: Broad-leaved evergreen; T-NL: Temperate needle-leaved; B-NL: Boreal needle-leaved.


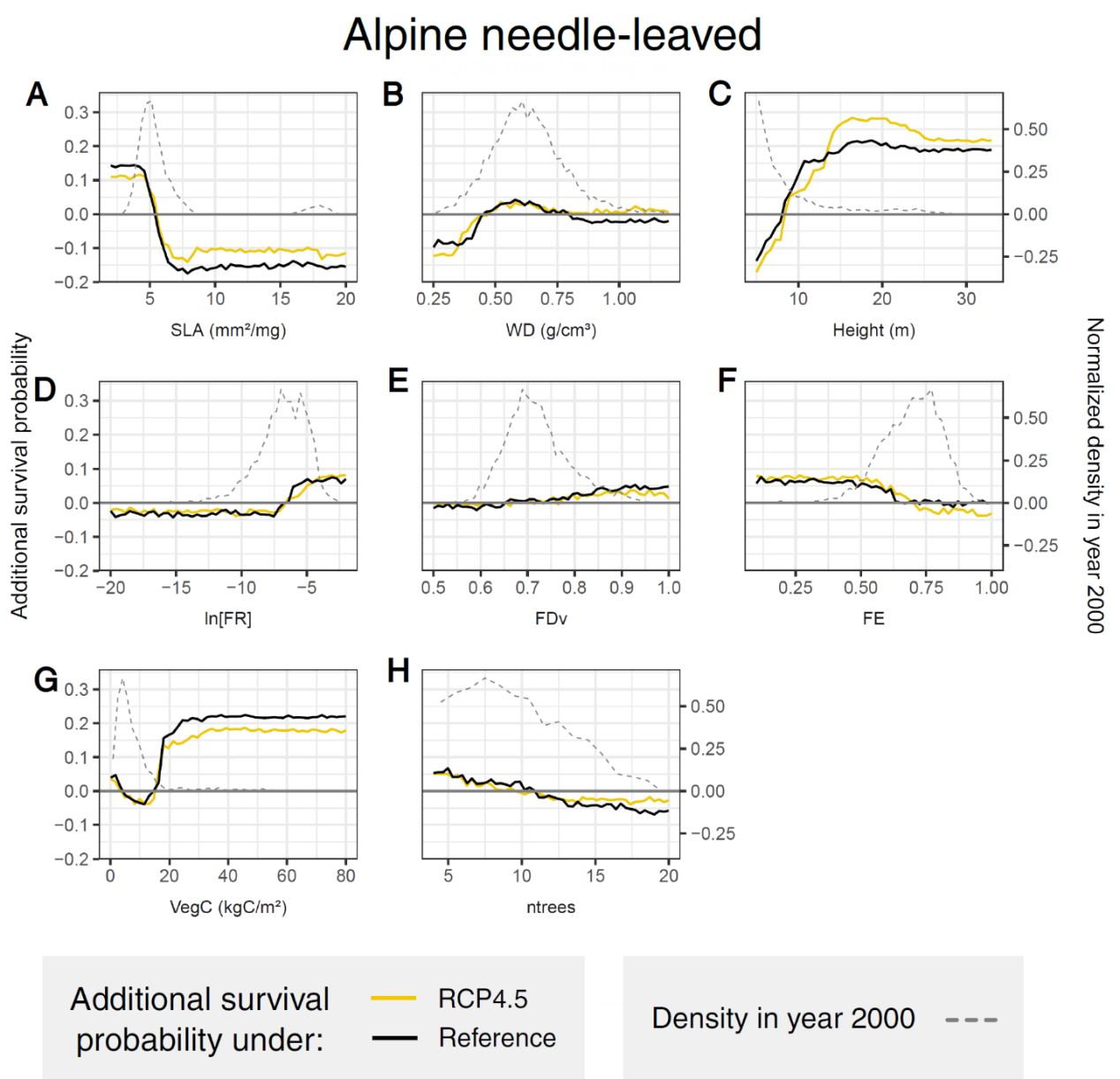


#### Supplementary Figure S6:

**Partial dependence plots in the alpine needle-leaved forest.** Dashed lines show initial distributions of the variables in year 2000. Solid lines show the additional mean survival probability of a randomly selected tree extracted from the random forest models. Values above zero indicate higher tree survival and vice versa. The x-axes show the respective variables. Variables: Specific leaf area (SLA), wood density (WD), tree height (Height), functional richness (FR), functional divergence (FDv), functional evenness (FE), vegetation carbon (VegC) and number of trees (ntrees).


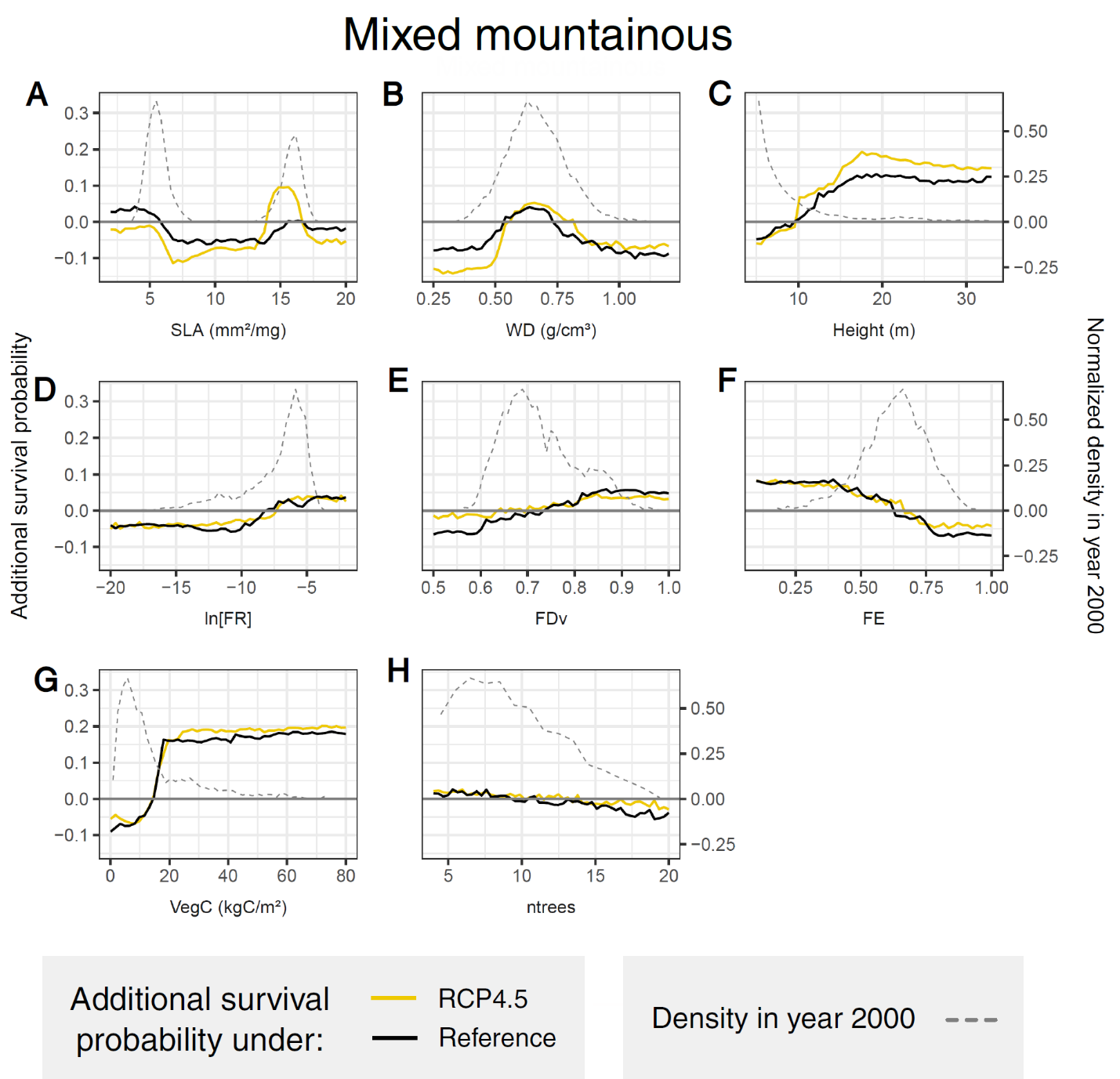


#### Supplementary Figure S7:

**Partial dependence plots in the mixed mountainous forest.** Dashed lines show initial distributions of the variables in year 2000. Solid lines show the additional mean survival probability of a randomly selected tree extracted from the random forest models. Values above zero indicate higher tree survival and vice versa. The x-axes show the respective variables. Variables: Specific leaf area (SLA), wood density (WD), tree height (Height), functional richness (FR), functional divergence (FDv), functional evenness (FE), vegetation carbon (VegC) and number of trees (ntrees).

####
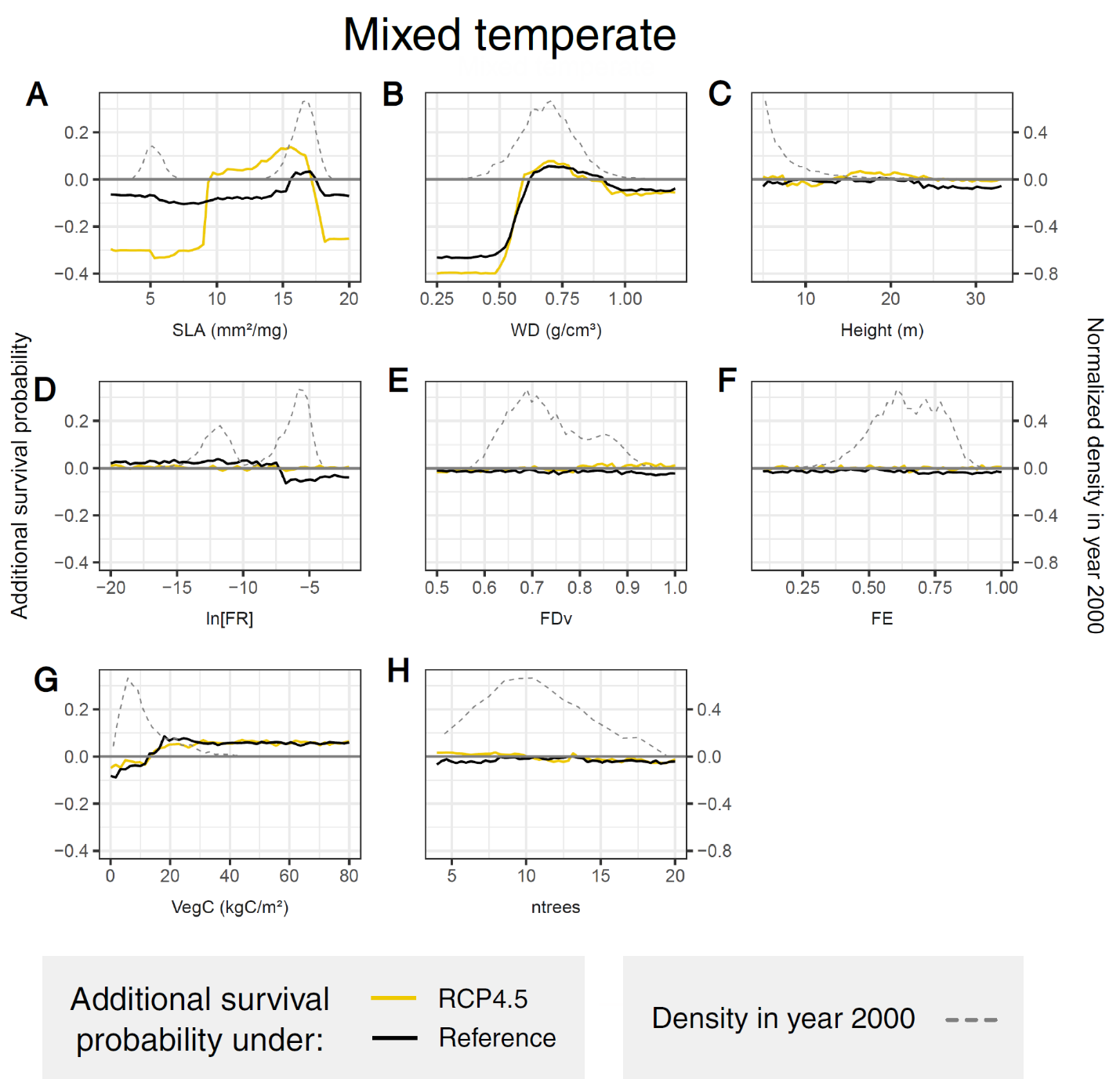
 Supplementary Figure S**8:**

**Partial dependence plots in the mixed temperate forest.** Dashed lines show initial distributions of the variables in year 2000. Solid lines show the additional mean survival probability of a randomly selected tree extracted from the random forest models. Values above zero indicate higher tree survival and vice versa. The x-axes show the respective variables. Variables: Specific leaf area (SLA), wood density (WD), tree height (Height), functional richness (FR), functional divergence (FDv), functional evenness (FE), vegetation carbon (VegC) and number of trees (ntrees).

####
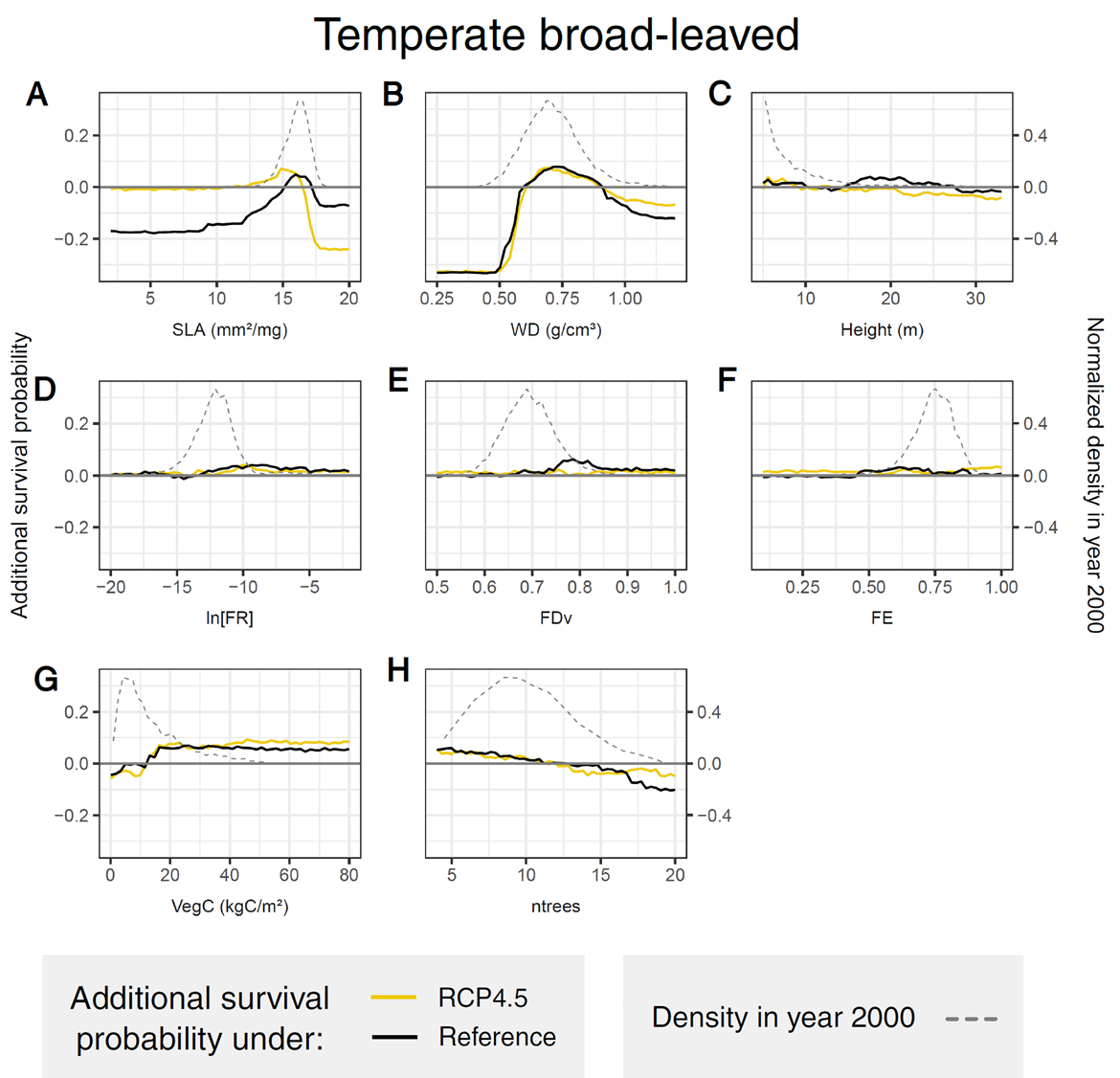
 Supplementary Figure S9:

**Partial dependence plots in the broad-leaved temperate forest.** Dashed lines show initial distributions of the variables in year 2000. Solid lines show the additional mean survival probability of a randomly selected tree extracted from the random forest models. Values above zero indicate higher tree survival and vice versa. The x-axes show the respective variables. Variables: Specific leaf area (SLA), wood density (WD), tree height (Height), functional richness (FR), functional divergence (FDv), functional evenness (FE), vegetation carbon (VegC) and number of trees (ntrees).

####
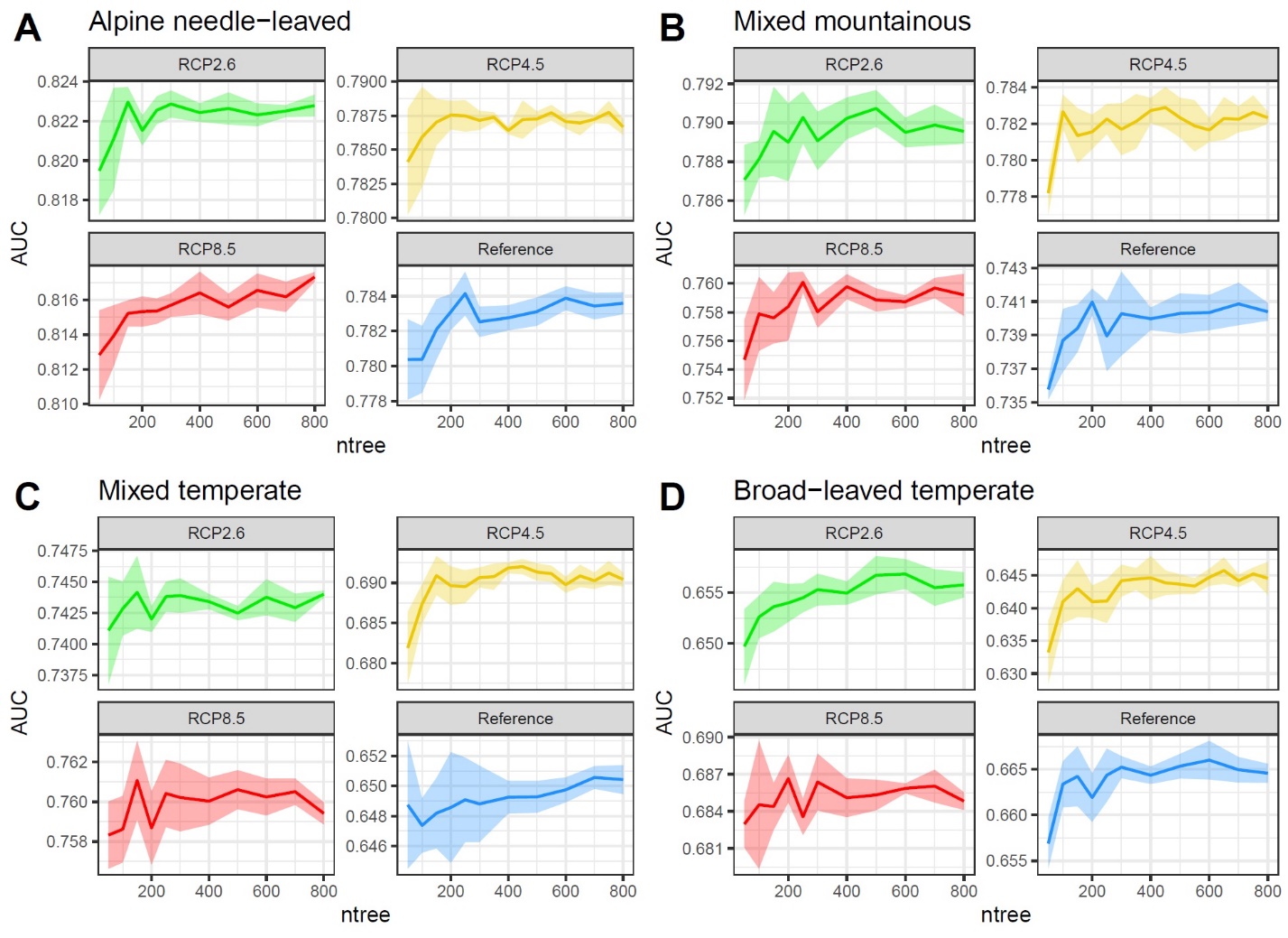
 Supplementary Figure S10:

**Parameter variation of ntree.** Variation of the hyperparameter ntree across all forest types and climate inputs. Model performance is evaluated with area under ROC curve (AUC) measure applied to the test data. The hyperparameter mtry was hold constant at 2. Envelops show the standard deviation and lines the arithmetic mean (n=5).

####
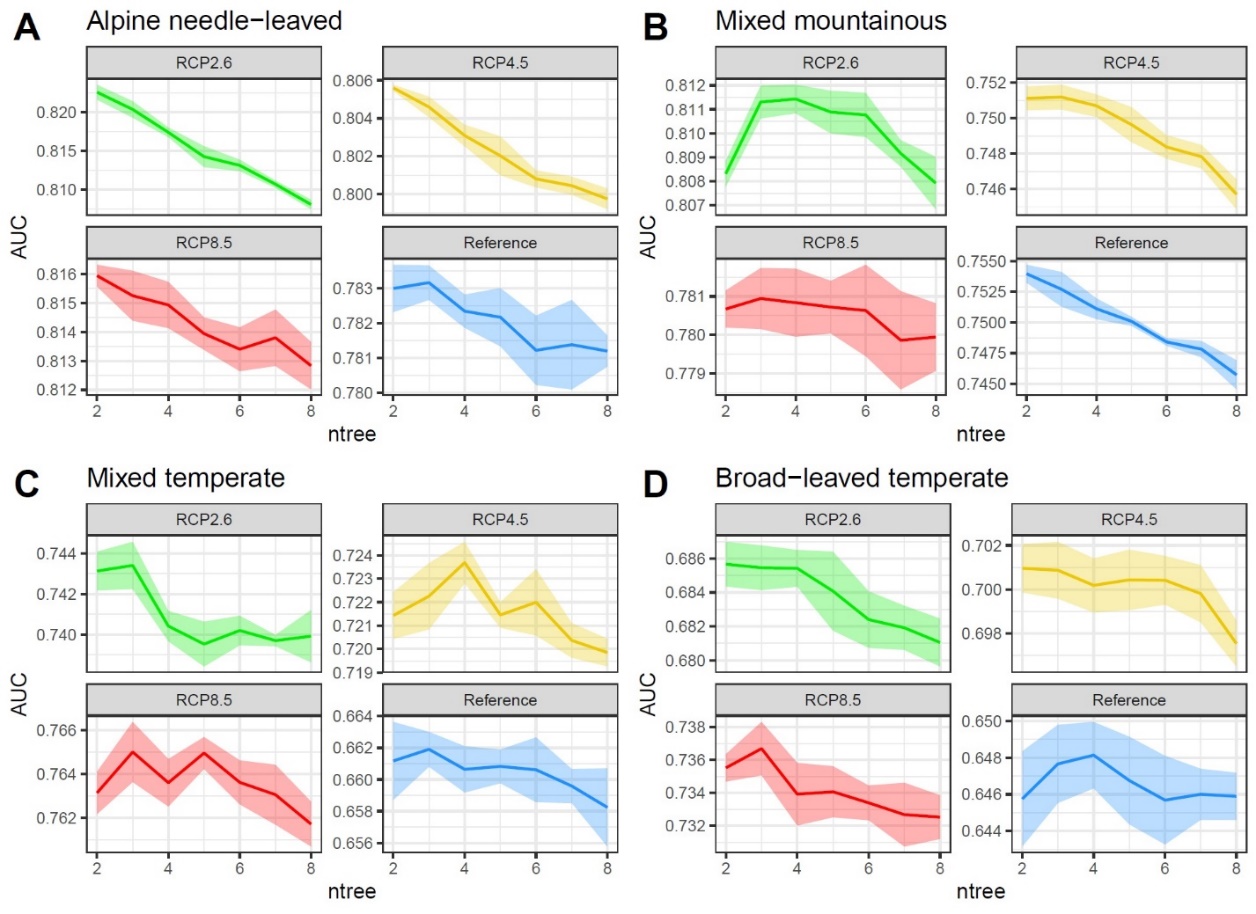
 Supplementary Figure S11:

**Parameter variation of mtry.** Variation of the hyperparameter mtry across all forest types and climate inputs. Model performance is evaluated with area under ROC curve (AUC) measure applied to the test data. The hyperparameter ntree was hold constant at 500. Envelops show the standard deviation and lines the arithmetic mean (n=5).


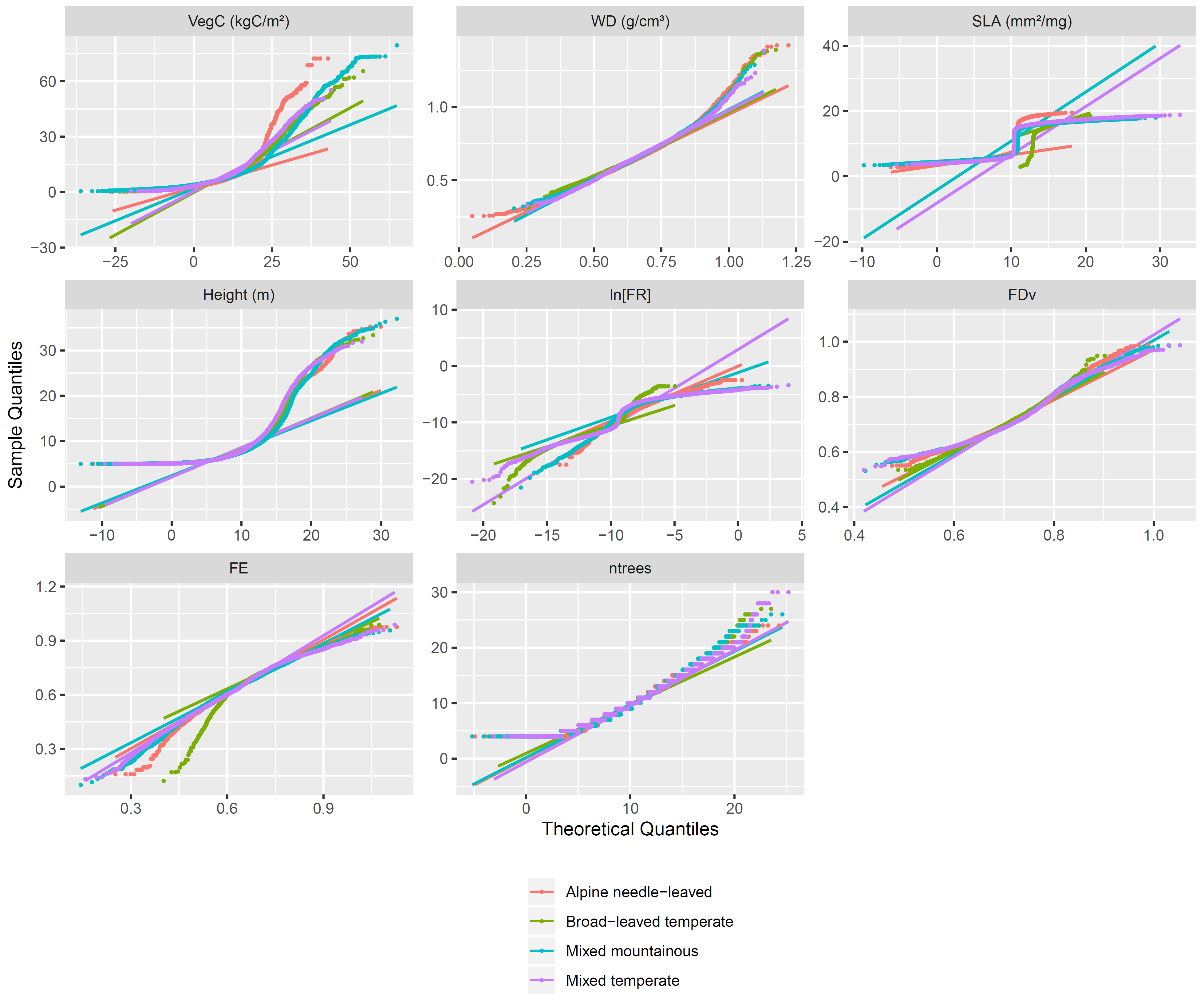


#### Supplementary Figure S12:

**QQ-Plots of training data statistics.** QQ-Plots for RCP4.5 across all forest types and for all variables: Specific leaf area (SLA), wood density (WD), tree height (Height), functional richness (FR), functional divergence (FDv), functional evenness (FE), vegetation carbon (VegC) and number of trees (ntrees).

#### Supplementary Figure S13:

**Mean annual mortality.** Simulated mean annual mortality of trees > 5m under reference climate depending on diameter at breast height (dbh).

#### Supplementary Figure S14:

**Mean annual mortality.** Simulated mean annual mortality of trees > 5m under reference climate depending on tree height.


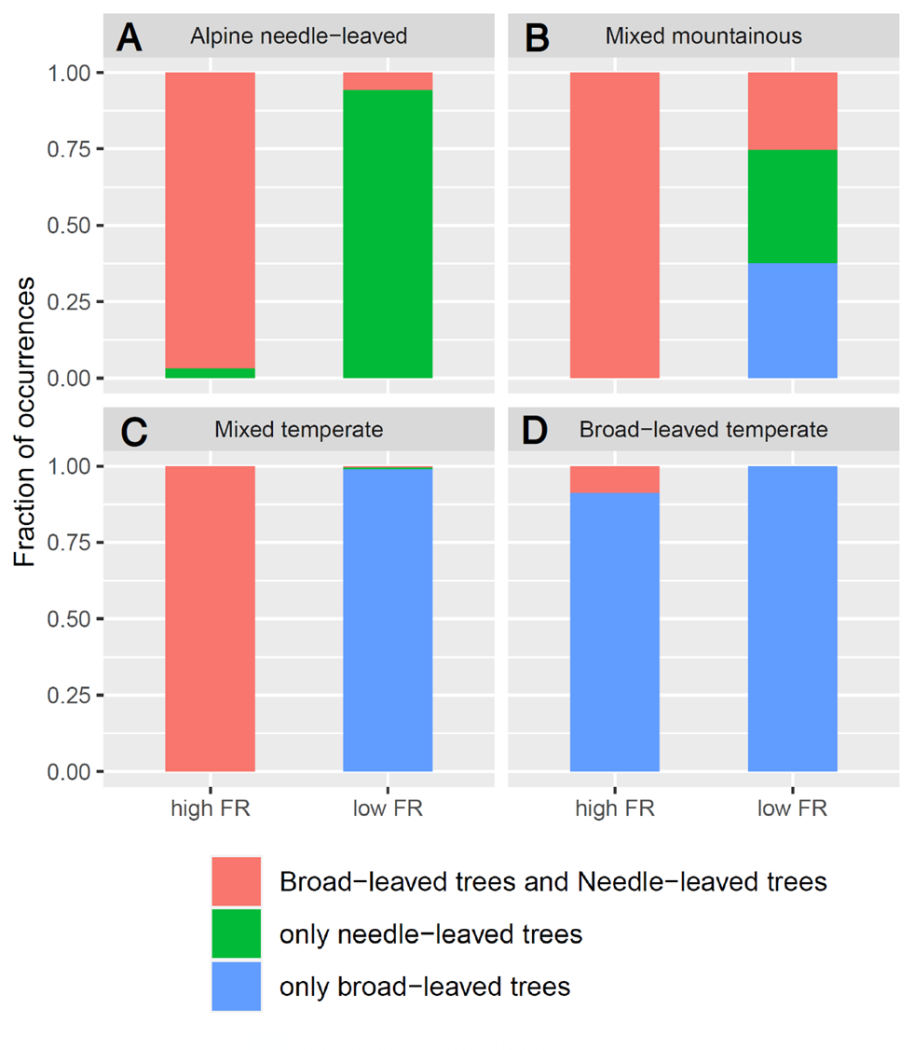


#### Supplementary Figure S15:

Occurrences of broad-leaved versus needle-leaved trees for each study site in year 2000 if functional richness is low (<25% percentile, right stack-bar in each panel) or high (>75% percentile, left stack-bar in each panel): Functional richness in forest patches is strongly characterized by the coexistence of broad- and needle-leaved trees (panel A-C), except for broad-leaved temperate forest (panel D).


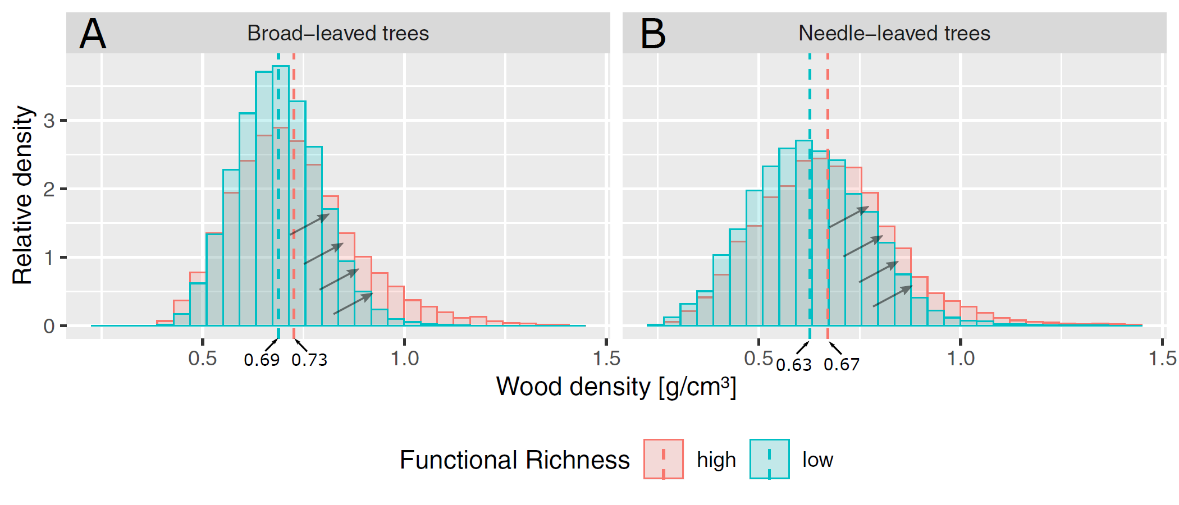


#### Supplementary Figure S16:

Wood density distributions averaged over all study sites in year 2000 for broad-leaved trees (Panel A) and needle-leaved trees (Panel B) if functional richness is low (<25% percentile, blue bars) or high (>75% percentile, red bars). Dashed lines indicate mean wood densities for each distribution and arrows illustrate the differences among distributions: Wood densities of broad- and needle-leaved trees are generally higher, if they grow on forest patches where both PFTs coexist and FR is high.


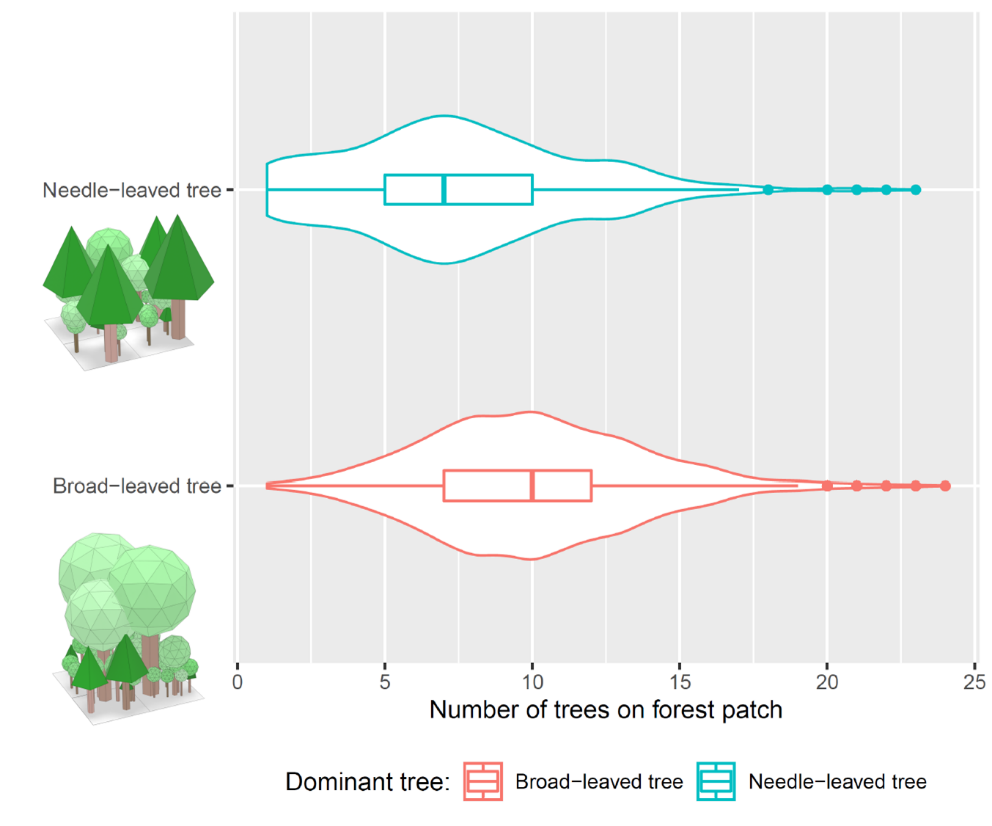


#### Supplementary Figure S17:

Local number of trees in the year 2000 in the mixed temperate forest, if the forest patch is dominated by either a single needle-leaved tree (top violin plot) or broad-leaved tree (>15m, bottom violin plot). Large needle-leaved trees generally suppress the growth of small trees due to their low SLA and high LAI. Illustrations show exemplary four forests patches in the year 2000 dominated by one of the two plant functional types in the temperate mixed forest site.


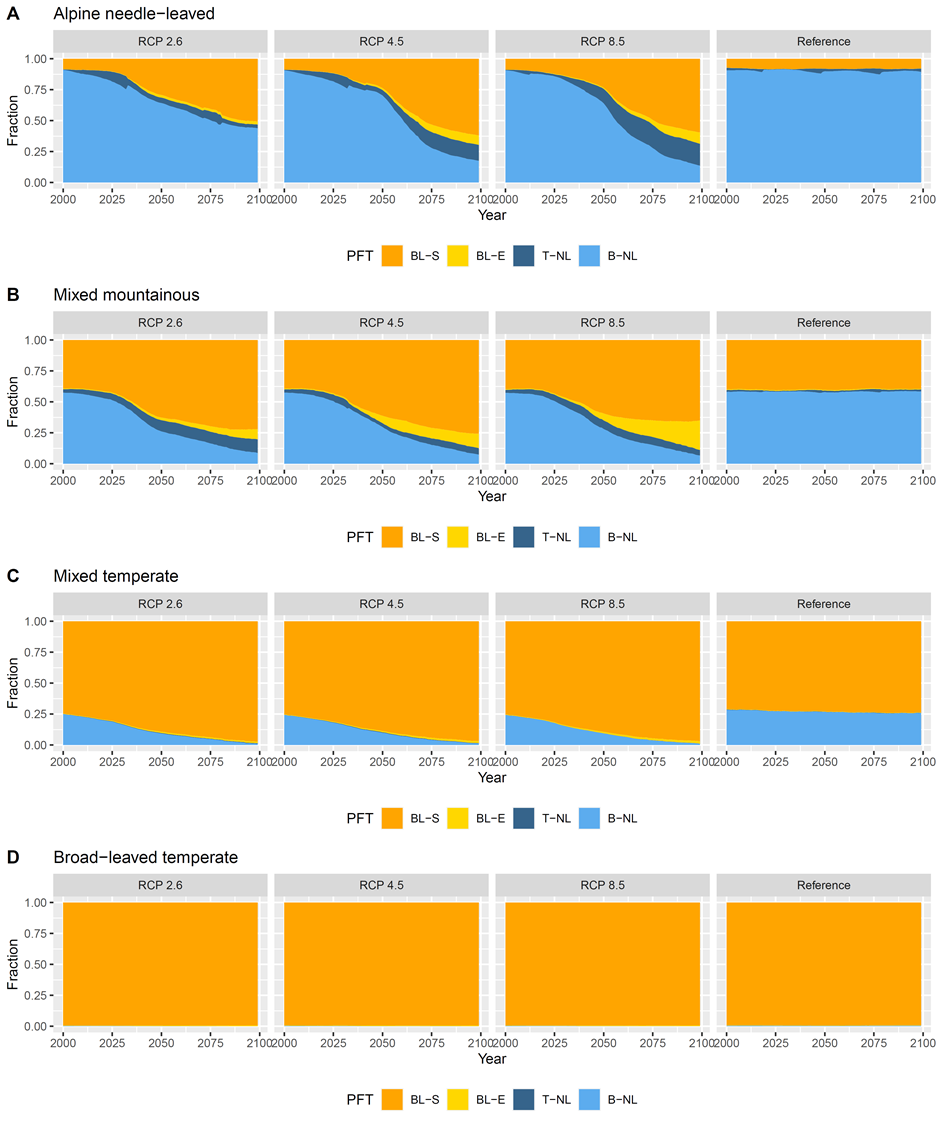


#### Supplementary Figure S18:

**Fraction of plant functional types (PFT).** Simulated fraction of all PFT across all forest types and input climates. RCPs and the reference climate between 2000 - 2099. BL-S: Broad-leaved summergreen; BL-E: Broad-leaved evergreen; T-NL: Temperate needle-leaved; B-NL: Boreal needle-leaved.


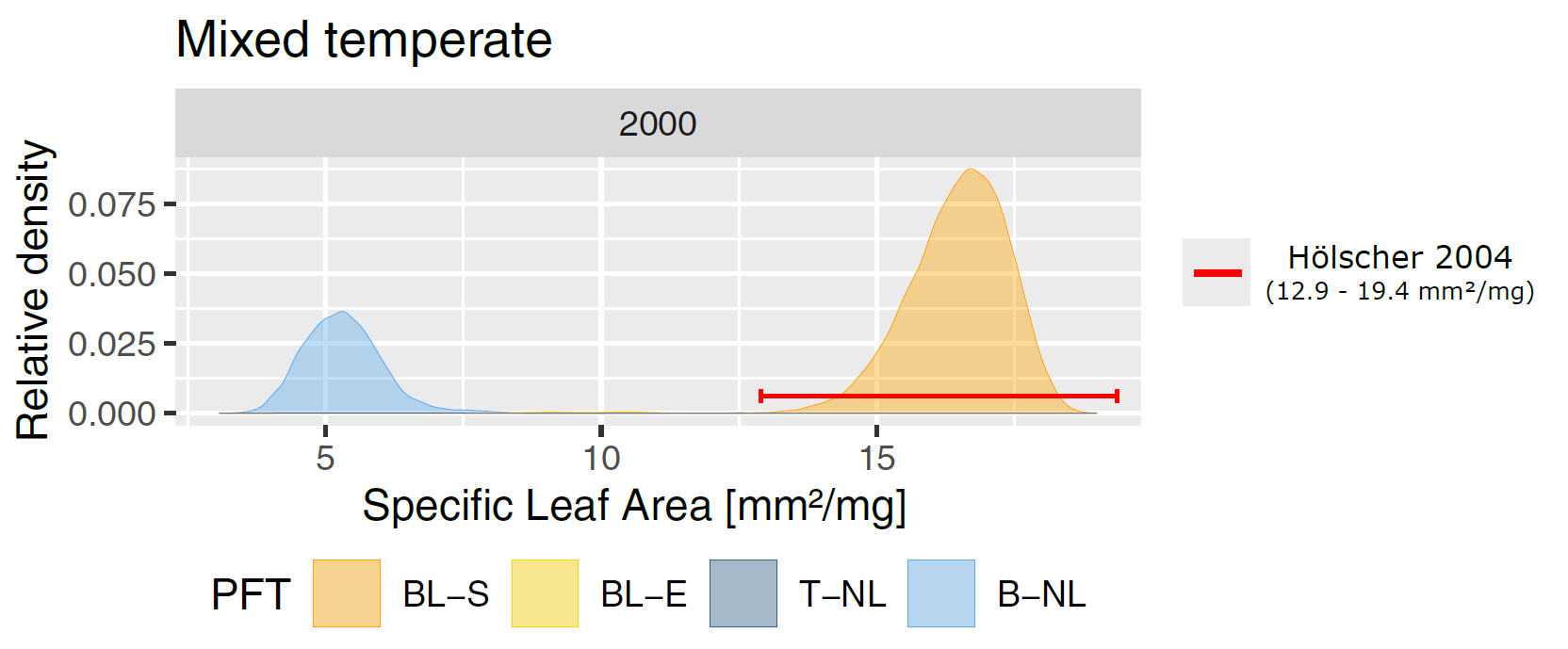


**Supplementary Figure S19:** Simulated SLA ranges in year 2000 at the Hainich NP of broad-leaved summergreen (BL-S), broadleaved evergreen (BL-E), temperate needle-leaved (T-NL) and boreal needle-leaved (B-NL) trees in comparison to observed ranges of dominant broad-leaved species in Hölscher (2004a).

**
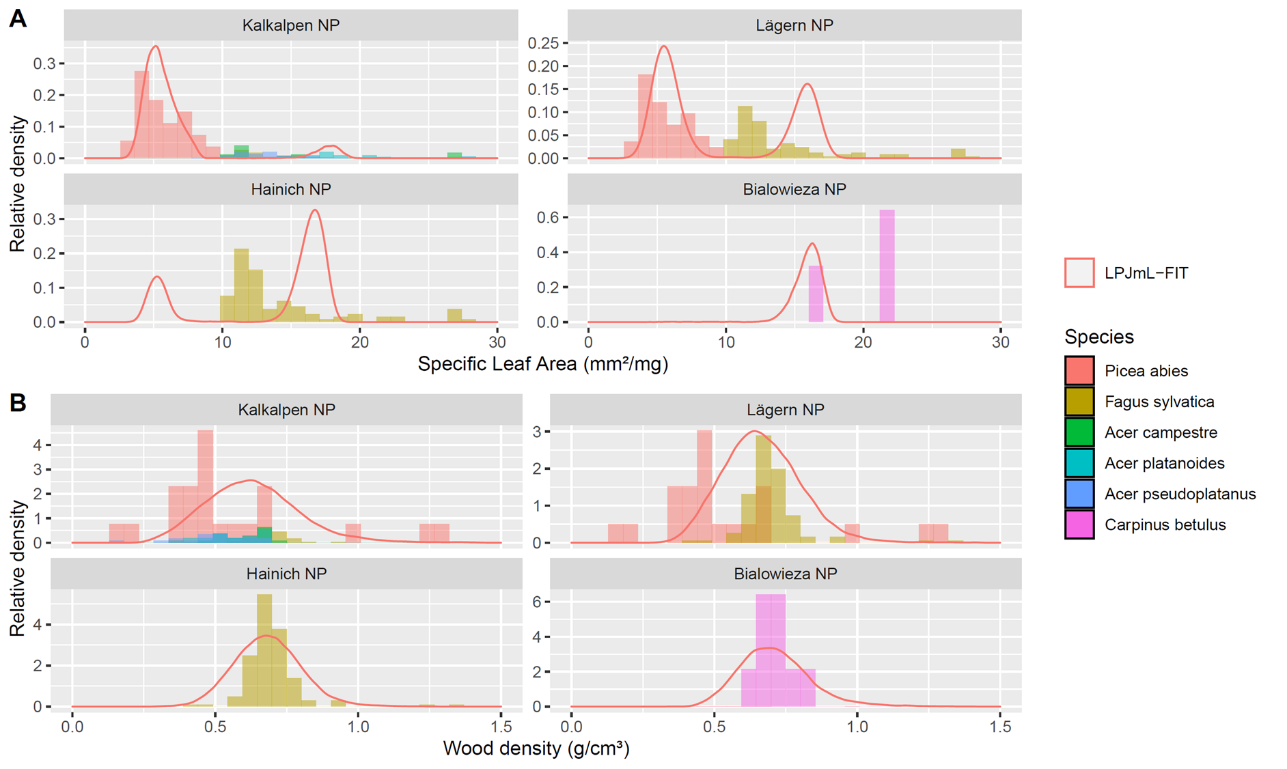
**

**Supplementary Figure S20:** Distribution of simulated specific leaf area (panel A) and wood density (panel B) compared to trait data according to the TRY database of dominant tree species observed at each study site (for dominant tree species observed see also Supplementary Table S1). Only georeferenced data entries in Europe (30 to 70 °N; -10 to 40°E) were used. Dominant tree species were weighted according to simulated fractions of broad- and needle-leaved trees to allow better comparability.


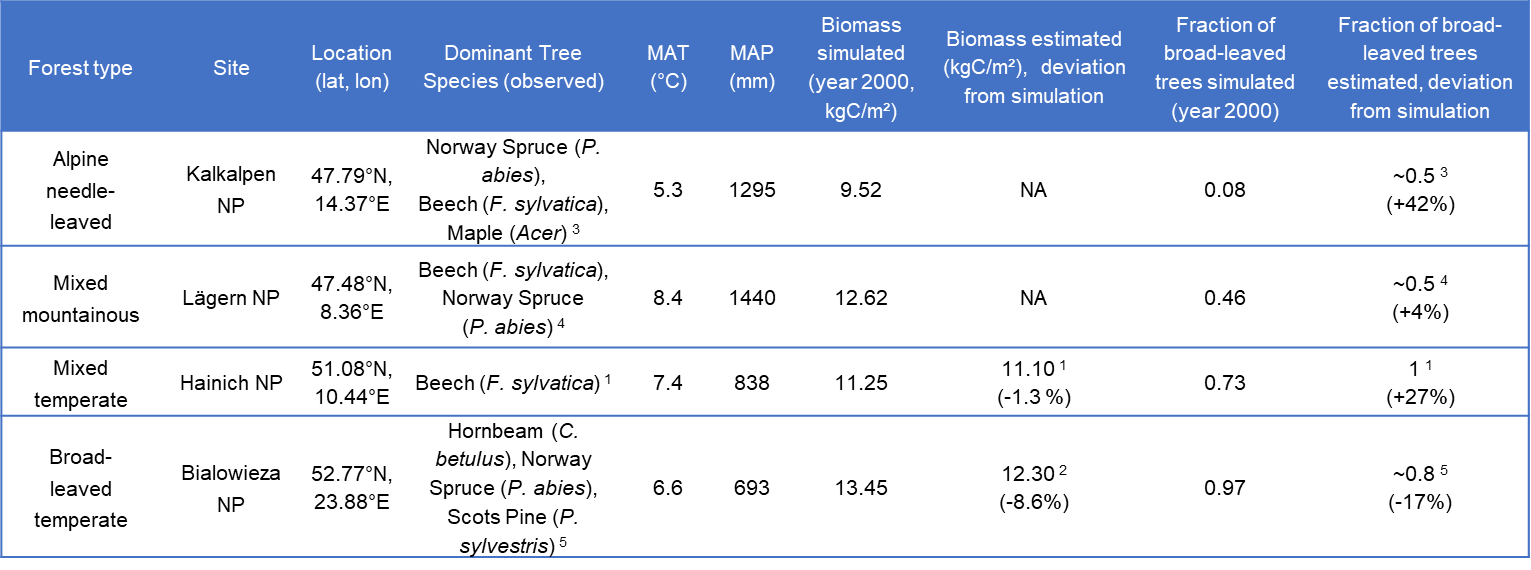


#### Supplementary Table S1:

**Forest type and site characteristics.** Four different forest types were used for LPJmL-FIT simulations. Climatic conditions were computed out of the climate input data. References for biomass and PFT fractions:

Hessenmöller et al. (2008)^1^, Matuszkiewicz et al. (2021)^2^, Flaschberger (2018)^3^, Schneider et al. (2017)^4^, Pawlaczyk (2009)^5^


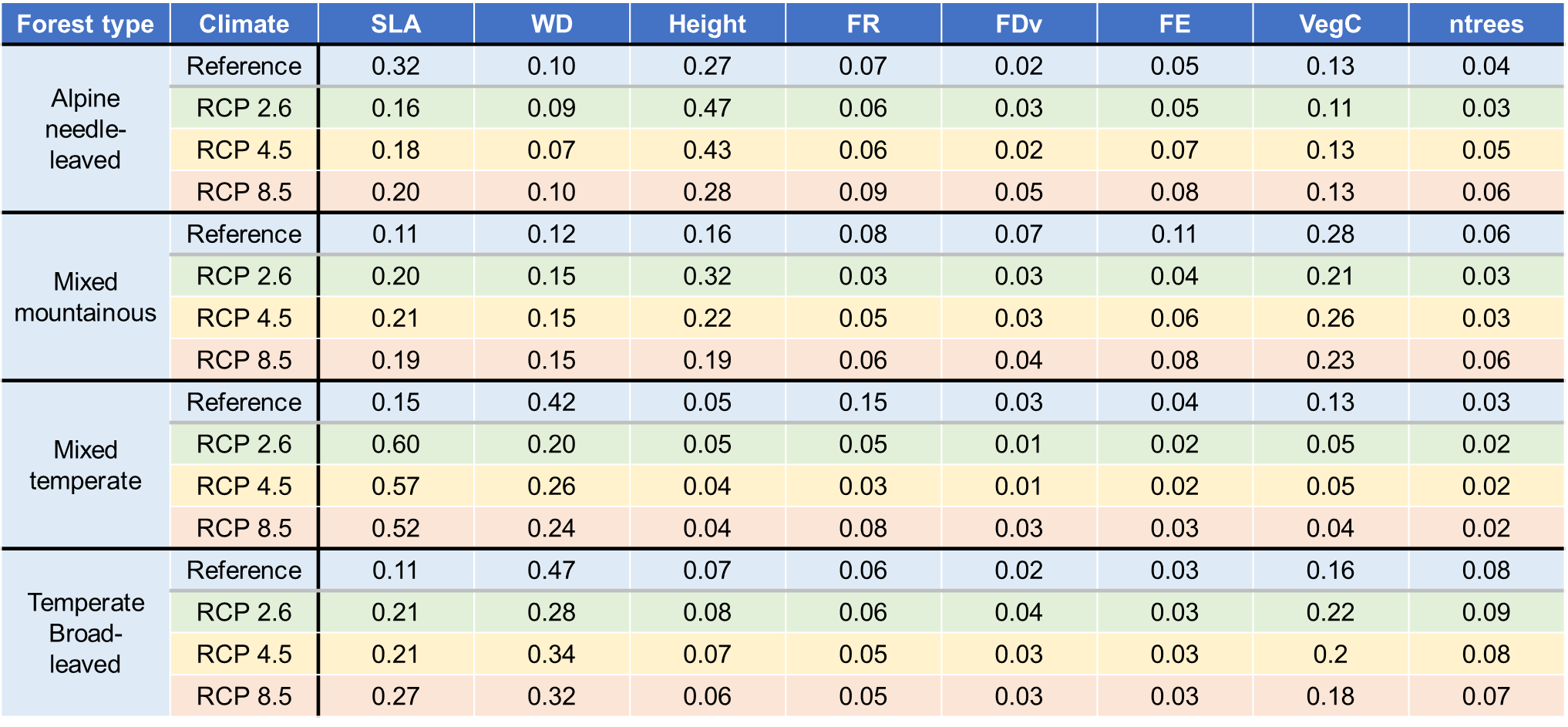


#### Supplementary Table S2:

**Summary of all variable importances.** Permutation variable importance across all forest types and inputs climates for all variables: Specific leaf area (SLA), wood density (WD), tree height (Height), functional richness (FR), functional divergence (FDv), functional evenness (FE), vegetation carbon (VegC) and number of trees (ntrees).

| **Forest type** | **Diversity Index** | **addition survival probability from FD (%)** | |
| --- | --- | --- | --- |
|  |  | Reference climate | Future climate |
| Alpine needle-leaved | FR | 6.72 ± 0.57 | 7.25 ± 1.05 |
|  | FDv | 3.78 ± 1.09 | 2.52 ± 0.76 |
|  | FE | 6.02 ± 0.77 | 7.07 ± 0.99 |
| Mixed mountainous | FR | 3.25 ± 0.86 | 3.35 ± 0.55 |
|  | FDv | 4.98 ± 0.83 | 3.47 ± 0.67 |
|  | FE | 7.21 ± 1.28 | 6.83 ± 0.44 |
| Mixed temperate | FR | -4.03 ± 0.77 | 0.33 ± 0.37 |
|  | FDv | -1.91 ± 0.64 | 1.17 ± 0.70 |
|  | FE | -1.12 ± 0.55 | 0.13 ± 0.61 |
| Broad-leaved temperate | FR | 2.80 ± 0.872 | 1.99 ± 0.77 |
|  | FDv | 3.23 ± 1.43 | 1.27 ± 0.58 |
|  | FE | 0.62 ± 1.35 | 1.58 ± 0.44 |

#### Supplementary Table S3:

**Survival probabilities from high FR, high FDv or low FE.** Survival probabilities correspond to mean (± standard deviation) of the partial dependence with high FR, high FDv and low FE of the upper/lower 10% percentiles of the initial distribution in Supplementary Figures S6 - S9, respectively.

#### Supplementary Table S4:

**Summary of training data statistics.** Mean, standard deviation, median, minimum and maximum for each forest types and all variables in the year 2000: Specific leaf area (SLA), wood density (WD), tree height (Height), functional richness (FR), functional divergence (FDv), functional evenness (FE), vegetation carbon (VegC) and number of trees on forest patches of 10 x 10 m (ntrees).


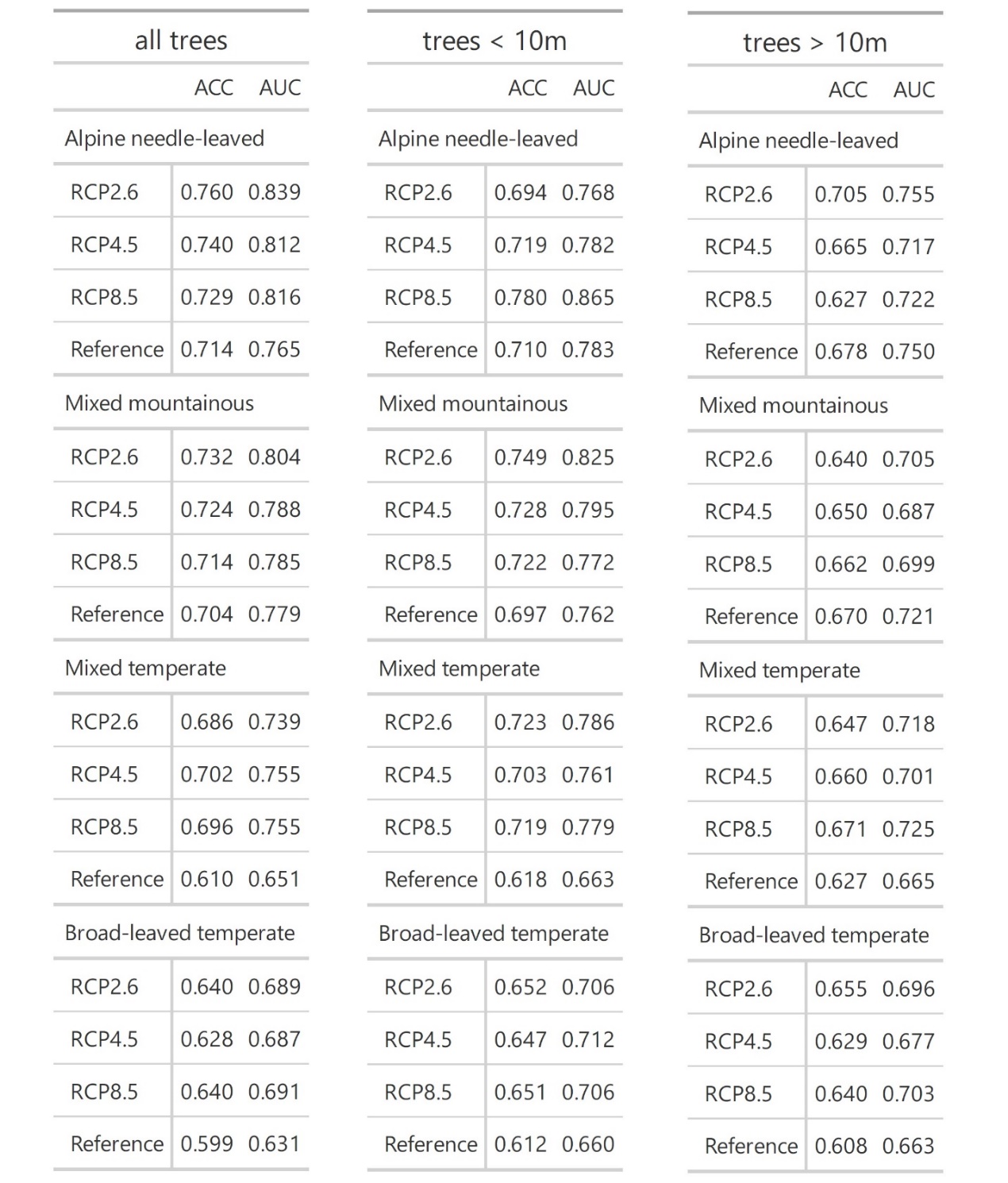


#### Supplementary Table S5:

**Summary of model evaluation.** Each random forest model was evaluated based on accuracy (ACC) and the area under the curve measure (AUC) in relation to the test data set.


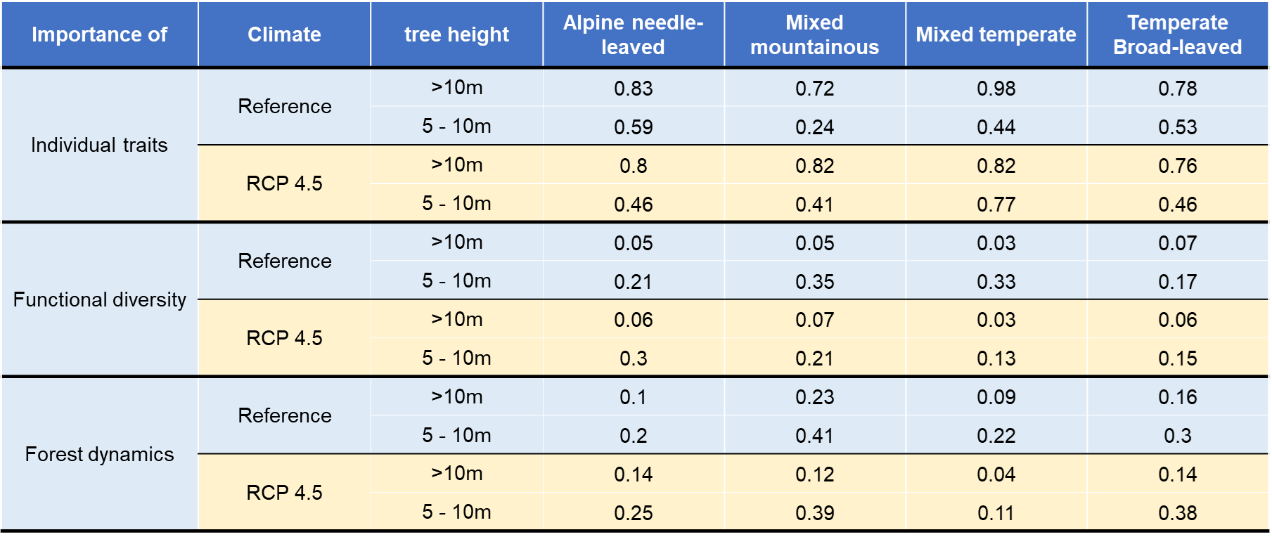


#### Supplementary Table S6:

Variable importance of individual functional traits, functional diversity and forest dynamic variables for each study site und reference and RCP4.5 separately for large trees (>10m) and small trees (between 5m – 10m). For all sites and climates functional diversity and forest dynamics are more important for small trees compared to large trees. Individual traits matter most for large trees consistently.

## Supplementary Methods

### Supplementary Methods A: Additional model description

The flexible trait-based vegetation model LPJmL-FIT simulates the establishment, competition, growth and mortality of individual trees which differ in their individual trait combinations. The model does not include forest management and simulates only natural forests. Trees grow on patches of sizes of 10m x 10m competing for light and water in each simulation step.

During establishment, a set of functional traits and the plant functional type is assigned randomly to each new tree. Traits are drawn randomly out of PFT-specific ranges derived from the global plant trait database TRY^6^, and are connected via trade-offs based on leaf and stem economics^7,8^. Functional traits (except tree height) remain fixed over the life time of a tree. Roots of each tree are distributed within the soil column according to a PFT-dependent distribution function and this distribution is constant over time^9^. Trees compete for water depending on their vertical distribution of fine roots and the amount of water present in each soil layer. Through resource competition and environmental filtering by local climate, only those trees survive that are best adapted to the local conditions. Over time, competitive and environmental filtering exclude trees with trait combinations that underperform under local conditions, leading trait distributions to converge^8^. Trees with trait combinations that maximize production and growth under local conditions are more likely to survive. Ultimately, the model includes a complex interplay of leaf and stem economics, individual production, competition and mortality - depending on local climate and community composition (Fig. 6).

In general, the model operates on the level of individual trees belonging to PFTs and not on pre-defined tree species. Although traits can vary within PFTs, drawing the connection to tree species has so far not been done. Currently, the model LPJmL-FIT can provide trait ranges/distributions and directions on how this can shift in the future. This approach makes the model particularly valuable for gaining new insights into tree competition, coexistence and functional trait ecology under the influence of a changing climate and stimulates the discussion on the importance of functional tree traits and their diversity for future.

### Supplementary Methods B: The influence of specific leaf area and wood density in the model

Specific leaf area and wood density are key traits to understand light competition and model performance. Specific leaf area determines all other leaf traits in the model, such as leaf longevity or leaf nitrogen, including a small uncertainty corridor^7^. In general, specific leaf area is anticorrelated to leaf longevity, nitrogen content per area and photosynthetic capacities of the leaves. Trees with high specific leaf area benefit from high carbon returns (high production compared to carbon investment), but have higher leaf turnover rates and a lower photosynthetic capacity^7^. Higher specific leaf area increases the leaf area index per carbon investment and thus shading of other trees. Therefore, specific leaf area has a strong influence on individual production and light competitiveness. Leaf phenology is determined by the plant functional type and depends on temperature, water stress and light availability^8^. For example, summergreen leaves are shed earlier in the year if temperature is too low, whereas leaves from needle-leaved trees can generally resist low temperatures.

Wood density influences individual growth and the growth-efficiency related mortality. Trees with high wood density need to invest more carbon into their stems to grow higher and therefore grow slower. On the other hand, high wood density lowers mortality related to maximum growth-efficiency, and thus the probability to die due to low annual performance^7^.

### Supplementary Methods C: Individual tree mortality

In LPJmL-FIT, tree mortality is composed of an age-dependent background mortality, and a growth-efficiency related mortality. The maximum of growth-efficiency related mortality is anti-correlated to the individual wood density of a tree, so that trees with higher WD have a lower probability to die if annual performance is low. The trade-off between WD and the maximum of growth-efficiency related mortality is implemented via an empirical function derived from global plant trait data^7,8^.

In this study, we observe a rather constant mortality rate for trees between 5m – 30m tree height, while mortality increases for trees larger than ~30m in almost every forest type (Supplementary Figure S13 and S14). The latter resembles other observed findings in natural forests^10,11^ and results from the increased age-dependent mortality for large trees in the model. In contrast to our simulations, however, current scientific literature typically observes clear U-shaped relationships between mortality and tree size^10–12^. Although, our model is currently missing a clear minimum for medium sized trees, we still observe a small dip for trees around 20m in our mortality patterns. Overall, mean simulated mortality rates range in between 1,2% and 2,2%, which agrees well with observed findings in other European natural forests (1.5% in Hainich NP in earlier publications^12^).

## Supplementary Discussion

### Supplementary Discussion A: Model mechanisms to explain complementarity effects

Our results show, that functional diversity affects tree survival differently and depends on local climate.

In the cold-limited alpine needle-leaved and mixed mountainous forest, positive effects of functional diversity emerge if functional leaf traits lay highly separated in trait space (high FR and FDv, low FE, see Table 1). Looking further into the simulated data, we observe that forest patches with low FR are usually dominated by one PFT (Supplementary Fig. S15, right-hand bars in each panel), whereas high FR arises from a distinct coexistence of broad- and needle-leaved trees (Supplementary Figure S15, shown in red in left bars of each panel).

Both plant functional types strongly differ in their specific leaf areas, leaf longevities and phenology, and thus in their annual variation of productivity functional leaf strategies. Needle-leaved trees are continuously foliated (high leaf longevity) and can fix carbon already early in the year when broad-leaved trees are still in progress of unfolding their leaves. On the other hand, broad-leaved trees are more productive than needle-leaved trees during warmer months. Due to this phenological complementarity, competition between broad- and needle-leaved trees is generally lower compared to the competition against individuals of the same PFT. Therefore, in case of coexistence, leaf and phenological complementarity generally reduces competition.

With less competition, trees with a higher wood density can thrive and stay in the tree community. Due to high wood density, these trees need to invest more carbon in their stem to grow and would be more likely to be excluded under stronger height-growth competition because of their slower overall growth. In the end, this effect generally increases the dominance of trees with higher wood densities in forest patches if functional diversity is high (Supplementary Figure S16, red bars), broadening the trait distribution. Trees with high wood density finally exhibit higher survival rate as higher wood densities reduce the overall mortality probability in the model. This explains higher tree survival in functionally diverse communities in the alpine needle-leaved and mixed mountainous forest in our model. Similar complementary effects in colder forests were also found in other studies^13^, where functional diversity increased the productivity of smaller trees in cold and temperate forests under current climate.

In the mixed temperate forest, high functional richness negatively affects survival under reference climate (Supplementary Figure S8D). Just as in the alpine needle-leaved and mixed mountainous forest, high functional richness emerges, if functional coexistence is given (Supplementary Figure S15, Panel C). However, due to the warmer climate, competition is generally on a higher level. Broad-leaved trees perform better, thereby challenging the survival of needle-leaved trees and which leads to a reduced survival of needle-leaved trees if they coexist with broad-leaved trees. Yet, due to stochasticity, some needle-leaved trees can survive understorey competition and growing up to the canopy and dominate single forest patches. Large needle-leaved trees can exhibit higher leaf area index (LAI) in the model, which allows them to effectively shade other understorey trees once they reach a certain tree height (see Supplementary Figure S17). The strong shading ability of large needle-leaved trees generally suppresses the growth of small trees including broad-leaved trees reducing their survival.

Therefore, we attribute the detrimental effect of functional richness in the mixed temperate forest to a mix of competitive exclusion and hierarchical competition^14,15^. Similar competitive effects were ascribed to hierarchical competition among trees in a temperate forest in China^16^.

### Supplementary Discussion B: Trait shifts observed under warming

Our simulations show that broad-leaved trees generally shift to lower SLA (Fig. 4). Those trait shifts arise from altered environmental filtering of young trees under warming: Over the century new individuals constantly join the tree community. These trees grow up under warmer climatic conditions than previous tree cohorts. Throughout environmental and competitive filtering, trees with different trait combinations survive better and finally form the future tree community, which causes trait distributions to shift.

Due to the implemented leaf economics in the model, trees with lower SLA generally have higher leaf longevities^7^. Those trees can shed their leaves later in the year and maintain higher production if vegetation periods become longer under warming climate conditions. Therefore, broad-leaved trees with lower SLA grow better under future climate, survive more often, which shifts SLA distributions to lower values. Similar trends of SLA were previously observed in response to increasing drought stress^17^.

### Supplementary Discussion C: Effects of RCPs on tree resistance and forest adaptation

Our results show, that the relative importance of functional traits remain rather similar across different climate change scenarios (Supplementary Table S2). This indicates that the mode of environmental filtering persists even under stronger warming. Simultaneously, complementarity effects might even become more decisive for tree survival with increasing climate severity, as the importance of functional diversity indices slightly increases in cold-limited forests under stronger warming scenarios (Supplementary Table S2).

Moreover, we did not detect abrupt changes of forest composition or vegetation carbon among the RCPs, that could indicate sudden broad-scale tree mortality events (Supplementary Figure S18 and Supplementary Figure S2). The composition and biomass of the forests develop gradually over time and did not display a collapse, as if forests would reach a tipping point. In the progress of forest succession, older, already existing trees die and make room for new trees with possibly better suited trait combinations to join the community. Those new trees grow better in a warmer environment and change the forest composition on decadal time scales (e.g. broad-leaved summergreen trees in Supplementary Figure S18). This hints to a relatively robust and gradually forest adaptation in functionally diverse and multi-aged forests, despite the reductions in biomass (Supplementary Figure 2) and changes in PFT composition (Supplementary Figure S18). The latter become more apparent the stronger climate change gets (compared to climate-change scenario RCP 8.5). Here, simulated forests biomass declines to ca. 8 kgC/m² until the end of the century across all forest types pointing towards more open forests where the proportion of needle-leaved trees decline in their foliar projective cover. It shows that the capacity of functionally diverse forests to resist climate change reaches its limits under strong climate warming (Supplementary Figure 1) towards the end of the 21^st^ century.

### Supplementary Discussion D: Site-specific model evaluation

Within this study, we initialized LPJmL-FIT at four different sites containing near-natural forest stands, which are located in the protected areas Kalkalpen (Austria), Laegern (Switzerland), Hainich (Germany) and Bialowieza (Poland). Those sites cover a wide range of central European climates to investigate biogeographic dynamics and forest resistance over a large climatic gradient.

The model has been extensively validated in earlier publications over a larger European domain regarding biomass, tree height and functional traits – and for gross primary production on site-specific level (same sites as in this study). In general, the validation of flexible-trait vegetation models remains challenging, because traits, aboveground biomass fluxes of carbon, water and energy are often not measured at the same site. So far, such holistic validation is only possible at very few sites. However, for a better comprehensibility and interpretation of the findings in this study, we reevaluated and validated our simulations with more site-specific data and discuss the representativeness of our simulated tree communities with observations in the following.

With respect to biomass, only two local estimates could be found at Hainich NP and Bialowieza NP. Here, the simulated values well agree with local estimates (deviations: 1,3% and 8,6%, see Table S1). Nevertheless, it would be desirable to obtain more data at the other sites as well to allow a more comprehensive evaluation.

The validation of functional traits remains challenging due to insufficient data situation. Site-specific trait records were found in Hainich^18^, but none could be found for the other sites of this study. However, at Hainich NP the simulated trait range (SLA) well matches the range of dominant broad-leaved tree species in the publication (Supplementary Figure S19). For further trait validation, we compared simulated trait distributions with trait ranges according to TRY of the dominant tree species observed at each study site (Supplementary Figure S20, for dominant tree species see Supplementary Table S1). Overall, simulated SLA ranges of needle-leaved trees well agree with observed ranges of *P. abies* in TRY (dominant tree species in Kalkalpen NP and Lägern NP). Simulated SLA ranges of broad-leaved trees are usually higher as for *F. sylvatica* in TRY, whereas wood density ranges simulated by LPJmL-FIT (red line, Supplementary Figure S20) mostly agree with TRY. As trait records of TRY were not sampled at the study sites, differences among TRY and LPJmL-FIT might emerge from trait plasticity and variability though local adaptation.

In this study, LPJmL-FIT simulates forests dominated by broad-leaved trees in **Bialowieza NP**, whereas observed tree communities vary from pure broad-leaved stands to mixed broad- and needle-leaved stands ^5^. Here, the spatial distribution of broadleaved tree vs. coniferous tree species strongly depends on soil moisture and soil water saturation at this site with coniferous species mostly occurring on marshy soils and bogs^5^. Since marshland and bogs have not yet been implemented in our model, we suggest that our results should be compared to forest stands growing on drained soils at this site.

In **Hainich NP**, our model simulates a fraction of 73% broad-leaved trees. However, the natural vegetation at this site comprises a mixture of only broad-leaved species dominated by F. sylvatica. Current studies suggest, that the observed dominance of F. sylvatica in primary forests such as Hainich NP, could be explained by the shade tolerance of juvenile trees ^19^. Therefore, we think that model deviations stem from the missing process of shade tolerance in the model. Including this process in the model might increase the fraction of broad-leaved trees reaching the canopy and lower the simulated fraction of needle-leaved trees. However, due to missing data, implementation of such processes is out-of-scope. Therefore, interpreting our results for beech dominated forest stands in Hainich NP must be seen in this context.

In **Lägern NP** the model simulates a mixed forest (fraction of broad-leaved trees: ~46%), which well agrees with observed fractions (~50%) ^4^. However, studies suggest that Lägern NP was once naturally dominated by F. sylvatica with few needle-leaved trees ^4^. Reproducing the dominant trait combinations in the range of F. sylvatica might be achievable by including a respective shade tolerance in the model.

In **Kalkalpen NP** the model simulates forests dominated by needle-leaved trees. Observed forest composition strongly varies within that protected area (from pure broad-leaved to pure needle-leaved stands) and strongly depends on the altitude^3^. To incorporate this altitudinal stratification would require high-resolution, i.e. downscaled climate scenario data, to force the model accordingly. Because such type of model forcings were not available in the project, our modelling results can be only compared to montane forest stands at this site with a MAT of about 5.3°C (as in Table S1).

The general focus of LPJmL-FIT is to simulate biogeographic dynamics based on environmental and competitive filtering. As the general approach, every tree growing strategy can establish in every grid cell at any time, while only environmental and competitive conditions determine which tree individuals survive over time and which do not. Within this framework, reproducing the exact trait and tree composition is not expected due to missing processes described above and path dependencies which very likely are also induced by historic human influences. Even if forest management dates back decades to centuries in some areas, it can still have some influence of forest composition and patch dynamics to date. Consequently, the representativeness of the simulated tree communities for site-specific vegetation must be taken with caution due to missing processes in the model and the ambiguity of former human influence.

However, we have chosen those sites because their forest dynamics are close to natural conditions and we wanted to test which climate and biodiversity factors in natural forests remain important to ensure future tree survival. Also, to give nature conservationist a scenario where further restoration allows the forest to develop into natural forests under climate change. To our knowledge, our study is the first to test how the importance of these factors would change under climate change and which role co-existence and competition of functionally different trees in the different tree layers would have. We are therefore convinced that our pure ecological approach makes the model particularly valuable for gaining new insights into tree competition, coexistence and functional trait ecology under the influence of a changing climate. It opens new paths to interpret how the local-specific processes under past, present and future conditions might modify our findings. We regard our study as a starting point which will stimulate the discussion on forest resistance on an ecological basis.

# References

1. Hessenmöller, D., Schulze, E. D. & Großmann, M. Bestandesentwicklung und Kohlenstoffspeicherung des Naturwaldes ‘Schönstedter Holz’ im Nationalpark Hainich. *Allg. Forst- und Jagdzeitung* **179**, 209–219 (2008).

2. Matuszkiewicz, J. M., Affek, A. N. & Kowalska, A. Current and potential carbon stock in the forest communities of the Białowieża Biosphere Reserve. *For. Ecol. Manage.* **502**, 119702 (2021).

3. Johann Flaschberger, J. K. *Waldkartierung Nationalpark Kalkalpen. Erfassung und Kartierung der Waldbestände, Waldentwicklungsphasen und Totholzsituation.* (Nationalpark Oö Kalkalpen GmbH, 2018).

4. Schneider, F. D. *et al.* Mapping functional diversity from remotely sensed morphological and physiological forest traits. *Nat. Commun.* **8**, 1–12 (2017).

5. Pawlaczyk, P. Forest communities. in *Białowieża National Park. Know it – Understand it – Protect it* (Białowieski Park Narodwy, 2009).

6. Kattge, J. *et al.* TRY - a global database of plant traits. *Glob. Chang. Biol.* **17**, 2905–2935 (2011).

7. Sakschewski, B. *et al.* Leaf and stem economics spectra drive diversity of functional plant traits in a dynamic global vegetation model. *Glob. Chang. Biol.* **21**, 2711–2725 (2015).

8. Thonicke, K. *et al.* Simulating functional diversity of European natural forests along climatic gradients. *J. Biogeogr.* **47**, 1069–1085 (2020).

9. Schaphoff, S. *et al.* LPJmL4 &amp;amp;ndash; a dynamic global vegetation model with managed land: Part I &amp;amp;ndash; Model description. *Geosci. Model Dev. Discuss.* 1–59 (2017) doi:10.5194/gmd-2017-145.

10. Monserud, R. A. & Sterba, H. Modeling individual tree mortality for Austrian forest species. *For. Ecol. Manage.* **113**, 109–123 (1999).

11. Hülsmann, L. *et al.* Does one model fit all? Patterns of beech mortality in natural forests of three European regions. *Ecol. Appl.* **26**, 2463–2477 (2016).

12. Holzwarth, F., Kahl, A., Bauhus, J. & Wirth, C. Many ways to die - partitioning tree mortality dynamics in a near-natural mixed deciduous forest. *J. Ecol.* **101**, 220–230 (2013).

13. Madrigal-González, J. *et al.* Complementarity effects on tree growth are contingent on tree size and climatic conditions across Europe. *Sci. Rep.* **6**, 1–10 (2016).

14. Kunstler, G. *et al.* Competitive interactions between forest trees are driven by species’ trait hierarchy, not phylogenetic or functional similarity: Implications for forest community assembly. *Ecol. Lett.* **15**, 831–840 (2012).

15. Jucker, T. *et al.* Competition for light and water play contrasting roles in driving diversity-productivity relationships in Iberian forests. *J. Ecol.* **102**, 1202–1213 (2014).

16. Pu, X., Umaña, M. N. & Jin, G. Trait-mediated neighbor effects on plant survival depend on life stages and stage-specific traits in a temperate forest. *For. Ecol. Manage.* **472**, 118250 (2020).

17. Greenwood, S. *et al.* Tree mortality across biomes is promoted by drought intensity, lower wood density and higher specific leaf area. *Ecol. Lett.* **20**, 539–553 (2017).

18. Hölscher, D. Leaf traits and photosynthetic parameters of saplings and adult trees of co-existing species in a temperate broad-leaved forest. *Basic Appl. Ecol.* **5**, 163–172 (2004).

19. Petrovska, R., Brang, P., Gessler, A., Bugmann, H. & Hobi, M. L. Grow slowly, persist, dominate—Explaining beech dominance in a primeval forest. *Ecol. Evol.* **11**, 10077–10089 (2021).
